# Surface functionalization affects the retention and bio-distribution of orally administered mesoporous silica nanoparticles in a colitis mouse model

**DOI:** 10.1038/s41598-023-47445-6

**Published:** 2023-11-17

**Authors:** Roman Schmid, Meta Volcic, Stephan Fischer, Zhi Qu, Holger Barth, Amirali Popat, Frank Kirchhoff, Mika Lindén

**Affiliations:** 1 Inorganic Chemistry II, Ulm University, 89081 Ulm, Germany; 2 Institute of Molecular Virology, Ulm University Medical Center, Ulm, Germany; 3 School of Pharmacy, The University of Queensland, Brisbane, QLD Australia; 4 Institute of Experimental and Clinical Pharmacology, and Toxicology and Pharmacology of Natural Products, Ulm University Medical Center, Ulm, Germany

**Keywords:** Materials chemistry, Diseases, Chemistry

## Abstract

Besides the many advantages of oral drug administration, challenges like premature drug degradation and limited bioavailability in the gastro-intestinal tract (GIT) remain. A prolonged residence time in the GIT is beneficial for enhancing the therapeutic outcome when treating diseases associated with an increased intestinal clearance rate, like inflammatory bowel disease (IBD). In this study, we synthesized rod-shaped mesoporous silica nanoparticles (MSNs) functionalized with polyethylene glycol (PEG) or hyaluronic acid (HA) and investigated their bio-distribution upon oral administration in vivo. The negatively charged, non-toxic particles showed different accumulation behavior over time in healthy mice and in mice with dextran sulfate sodium (DSS)-induced intestinal inflammation. PEGylated particles were shown to accumulate in the lower intestinal tract of healthy animals, whereas inflammation promoted retention of HA-functionalized particles in this area. Overall systemic absorption was low. However, some particles were detected in organs of mice with DSS-induced colitis, especially in the case of MSN-PEG. The in vivo findings were connected to surface chemistry-related differences in particle adhesion on Caco-2/Raji and mucus-producing Caco-2/Raji/HT29 cell co-culture epithelial models in vitro. While the particle adhesion behavior in vivo was mirrored in the in vitro results, this was not the case for the resorption results, suggesting that the in vitro model does not fully reflect the erosion of the inflamed epithelial tissue. Overall, our study demonstrates the possibility to modulate accumulation and retention of MSNs in the GIT of mice with and without inflammation through surface functionalization, which has important implications for the formulation of nanoparticle-based delivery systems for oral delivery applications.

## Introduction

Oral drug delivery offers many advantages over other administration routes in terms of patient compliance, cost effectiveness, and avoidance of infection and pain. However, oral delivery is also challenging, especially when it comes to administration of macromolecular drugs, due to the harsh conditions prevailing in the GIT, like varying pH, and enzymatic activity. Additional challenges include reduced exposure of cells to the therapeutics due to shielding through the mucosal barrier and consequently fast transportation off-site along with intestinal contents resulting in low overall bioavailability^1–3^. Approaches to overcome the latter include the development of long-lasting drug delivery platforms accumulating at the intestinal wall^4^. Such delivery platforms may be based on mesoporous silica nano- or microparticles, which offer distinct benefits due to their high porosity and specific surface area, as well as their tunable size, shape and charge. Especially interesting is their straightforward surface functionalization. With the aim to increase their oral bio-availability, mesoporous silica particles (MSNs) have been loaded with a variety of therapeutics, for example insulin^5,6^, budesonide^7^, 5-amino salicylic acid^8^, paclitaxel^9^, antitubercular agents^10^, antibiotics^11^ or notch inhibitors^12^. In general, silica is considered to be safe by the US Food and Drug Administration (FDA) and the European Food Safety Authority (EFSA)^13^ and oral administration of mesoporous silica particles at doses of 9 g per day was well tolerated in human trials^14,15^.

A pre-requisite for accumulation at the intestinal epithelium is that the carrier itself should show a pro-longed retention in the GIT, and that transportation off-site either via intestinal absorption or via excretion along with the luminal contents is low. A previous study by Li et al.^16^ examined how the shape of MSNs influences their bio-distribution upon oral administration. Here, negatively charged, all-silica particles with different aspect ratios (AR) of 1 (= spherical particles), 1.75 and 5 and a particle length of 85 nm, 146 nm and 483 nm, respectively, were examined. The particles were absorbed into systemic circulation already after 2 h and were detected in defense organs (spleen and liver) up to 72 h whereas the elongated particles (AR 5) were overall less abundant in internal organs over the time periods studied. Furthermore, the spherical particles were excreted both via feces and the urine, while elongated particles were almost exclusively cleared through the feces, and at a rate which was slower than that of the spherical counterparts. Similar results were reported by Zhao et al*.*^17^, where systemic absorption and accumulation in defense organs was pronounced for spherical particles, and these exhibited shorter circulation times as compared to long-rod MSNs (AR 4). 7 days post-administration, concentrations of elongated MSNs (AR 4) were still elevated in the organs and in the feces. The same study reported that elongated MSNs were more retained in the colon at 12 h post-administration as compared to spherical particles. Overall, systemic absorption of rod-like particles seemed to be low, but at the same time, residence times in the GIT were prolonged.

One major obstacle a drug transporter encounters on its path to the epithelium is the mucus barrier of which the main component is glycosylated mucin-2 proteins forming a dense hexagonal mesh-like hydrogel. These mucus layers act as natural diffusion barrier against pathogens, bacteria as well as drug carriers leading to their entrapment and fast clearance from the GIT^18,19^. An important factor influencing the interaction of potential drug carriers with the mucus barrier and therefore their availability at the intestinal wall is their size. The ability of particles to diffuse through mucus is inversely correlated with their size, and hardly occurs for particles having diameters exceeding 200 nm^20,21^.

Another key parameter influencing the interaction of nanoparticles with mucus is their surface chemistries. For example, it is well known that surface functionalization with PEG reduces particle-mucin interactions, therefore enabling the particles to penetrate easily through the mucus^22–24^. The effect is more pronounced for high PEG packing densities, and can be affirmed over a wide molecular weight range^25–27^. On the other hand, mucus-adhesiveness is characterized by strong interactions with mucins, a property well-known for polymers like chitosan^2^. However, other than the term suggests, mucus-adhesiveness might actually be counteractive when it comes to delivery of therapeutics towards the healthy intestinal epithelium^19^. Mucus-adhesive particles are unlikely to penetrate through the mucosal barrier towards the epithelium, and are therefore more prone to be transported off-site due to rapid mucus turnover rates^28^. A comparative study of the translocation of PLGA micro- and nanoparticles, either coated with mucus-adhesive chitosan or mucus-penetrating PEG, across colonic tissue ex vivo, demonstrated that chitosan coated particles did not enter the mucosa, but negatively charged PEGylated particles did, and were eventually found translocated across the mucosa^29^. Increased translocation and permeation in vivo has also been reported for positively charged PEGylated MSNs loaded with PEI-coated carbon dots^30^.

In vitro models of the intestinal epithelium are widely used to study toxic effects on epithelial integrity, drug permeation or internalization of drug carriers. These models are typically comprised of a dense Caco-2 cell layer cultivated on permeable membranes and can be further refined by introducing additional cell lines as co-cultures, such as mucus producing HT29 cells, as well as Raji cells in order to promote differentiation into M-cells. This results in triple-co-cultures closely mimicking the intact intestinal epithelium in a healthy state^31–37^. However, the intactness of the intestinal mucosa can be affected by a variety of diseases, especially in the case of IBD. IBD is a generic term for Crohn’s disease and ulcerative colitis, both resulting in chronic inflammation of the GIT and disruption of the mucosal integrity followed by infiltration of immune cells and pathogens, which typically results in symptoms like diarrhea, weight loss, abdominal pain and ulceration^38^. While Crohn’s disease can affect the whole GIT, inflammation and ulceration in ulcerative colitis is mostly limited to the lower parts of the GIT^39^. Additionally, ulcerative colitis is accompanied with faster colonic transit times^40^, pronouncing the urge for the design of a drug delivery platform exhibiting accumulation and prolonged retention at the intestinal wall. While the healthy intestinal epithelium may be modelled in vitro to some extent, in vivo models are widely used to mimic the conditions present in IBD. A well-established in vivo model to reassemble the clinical picture of ulcerative colitis is dextran sulfate sodium (DSS)-induced colitis, which results in reproducible alteration of the mucosal barrier, as well as in the formation of inflamed lesions caused by infiltration of bacteria and recruitment of immune cells, such as macrophages and granulocytes^41,42^. Promising drug release profiles in DSS-induced colitis in mice with MSNs surface functionalized with the anionic, carboxylic acid group-containing hydrophilic polymer Eudragit S100 and loaded with Budesonide were recently demonstrated by Qu et al.^7^. Distribution and retention of silica particles with sizes of 200–300 nm after a single oral dose in healthy and DSS-treated mice was reported by Tam et al.^43^. The particles reached the distal colon, but no differences in accumulation between the DSS and control groups were observed, reinforcing the need for particle modifications which increase retention times in inflamed GIT. Furthermore, inflamed GIT regions are reported to be rich in cationic proteins^44,45^, and anionic liposomes are reported to adhere stronger in inflamed colonic mucosa than their cationic counterparts^46^. HA is a negatively charged bio-polymer which has been shown to potentially accumulate in GIT under inflammatory conditions when used in rather complex multi-stage co-formulation approaches^47,48^.

Encouraged by these findings, this work aimed at the comprehensive, direct comparison of mucus-penetrative or inflammation-targeted MSNs functionalized with either PEG or HA via well-established synthesis protocols and the influence of these polymers on the intestinal and organ-specific bio-distribution in vivo upon oral administration under both, healthy and inflammatory conditions. In order to increase their retention time, rod-shaped MSNs were synthesized exhibiting an AR of 3. The length of the particles was kept below 200 nm to enable potential penetration through the mucosal mesh. Bio-distribution of MSNs in GIT and organs were investigated via fluorescence imaging after oral gavage of a single dose and was connected to particle accumulation on cell-based epithelial models in the presence and absence of mucus producing cells.

## Experimental section

### Chemicals and materials

Tetraethyl orthosilicate (TEOS), (3-aminopropyl) triethoxysilane (APTES), dry toluene, *N*-(3-dimethylaminopropyl)-*N*′-ethylcarbodiimide hydrochloride (EDC), N-hydroxysuccinimide (NHS), hyaluronic acid sodium salt from *Streptococcus Equi* (HA, 1.5–1.8 × 10^3^ kDa), low molecular weight chitosan (50–190 kDa, 75–85% deacetylated), triethylamine, 3-(triethoxysilyl) propyl isocyanate, dichloromethane, IL-1β and LPS were purchased from Sigma-Aldrich Chemie GmbH, Schnelldorf, Germany. Cetyltrimethylammonium bromide (CTAB), ammonium hydroxide (28% and 32% in water), sodium hydroxide, acetic acid, methanol, ethanol, acetone and ethylene glycol were purchased from VWR International GmbH, Darmstadt, Germany. Ammonium nitrate and dialysis tubing were purchased from Carl Roth GmbH & Co. KG, Karlsruhe, Germany. (4-(2-hydroxyethyl)-1-piperazineethanesulfonic acid) (HEPES), sodium taurocholate, sodium chloride, l-α-lecithin (Egg Yolk), Immobilon-FL PVDF membranes and disodium maleate were purchased from Merck KgaA, Darmstadt, Germany. Cy5-NHS was purchased from Lumiprobe GmbH, Hannover, Germany. DMSO (amine free), Phosphate buffered saline (PBS), BCA protein assay kit and N-hydroxysulfosuccinimide (sulfo-NHS) were purchased from Thermo Fisher Scientific, Karlsruhe, Germany. ATTO647N-NHS, ATTO647N-NH2 and ATTO565-NHS were purchased from ATTO-TEC GmbH, Siegen, Germany. ɑ-methoxy-ω-NHS polyethylene glycol with a molecular weight of 5056 Da (PEG_5k_) was purchased from Rapp Polymere, Tübingen, Germany. Human colon adenocarcinoma Caco-2 cells (Acc No. 86010202), human colon HT29-MTX-E12 cells (Acc No. 12040401) and human lymph Raji cells (Acc No: 85011429) cells were obtained from the European Collection of Authenticated Cell Cultures (ECACC), Porton Down, United Kingdom. DMEM, RPMI, fetal bovine serum (FBS) and MEM non-essential amino acid solution were purchased from Gibco, Carlsbad, USA. l-glutamine and penicillin–streptomycin solutions were purchased from PAN-Biotech GmbH, Aidenbach, Germany. 0.4 µm 12 well PET TC-Inserts were purchased from Sarstedt AG & Co. KG, Nümbrecht, Germany. Alexa Fluor^TM^488-Phalloidin was purchased from Invitrogen, Carlsbad, USA. 96-well plates were purchased from Corning Incorporated, New York, USA. CellTiter 96^®^ AQ_ueous_ One Solution Cell Proliferation Assay (MTS) was purchased from Promega, Fitchburg, USA. Fluorescence-free diet Teklad Global Diet 2019S was purchased from Inotiv, Lafayette, USA. Lysis buffer (50 mM Tris pH 7,4, 150 mM NaCl, 2 mM EGTA, 2 mM EDTA, 25 mM NaF, 25 mM β-glycerophosphate, 0.1 mM NaV and proteinase inhibitor) was purchased from Roche, Mannheim, Germany. Protein sample loading buffer was purchased from Li-COR, Lincoln, USA. Anti-CD44 ab254530 Abcam and anti-actin ab8227 were purchased from Abcam, Cambridge, United Kingdom.

### Synthesis of elongated, rod-shaped mesoporous silica nanoparticles

Elongated mesoporous silica nanoparticles with an aspect ratio of 2.7 were synthesized according to a modified synthesis of that originally reported by Huang et al*.*^49^ CTAB (2.2 g, 6 mmol) was dissolved in a mixture of water (285 mL), ammonium hydroxide solution (32 wt%, 16.6 M, 5.9 mL, 98 mmol) and ethylene glycol (27.8 mL, 0.4 mol) in a 0.5 L round-bottomed flask at 60 °C. After cooling to room temperature (RT), TEOS (4.7 mL, 21.2 mmol) was added to the mixture at a stirring rate of 600 rpm and the reaction proceeded for 4 h. The particles were precipitated with ammonium nitrate (30 g, 23.5 mmol), separated via centrifugation and thoroughly washed with ethanol twice and acetone once in an ultrasonic bath for 1 h each. After drying at 60 °C in vacuo, the particles were calcined at 550 °C for 7 h (heating rate 1.5°C min^−1^) yielding all-silica, rod-shaped mesoporous silica nanoparticles, entitled “MSN-c”.

### Post-functionalization of MSNs with amino groups

Calcined “MSN-c” were dried at 100 °C in vacuo overnight. After dispersing the particles (300 mg, 4.6 mg mL^−1^) in dry toluene using a focused ultrasonic bath (Covaris S220) the dispersion was transferred in a 0.25 L round-bottomed flask and stirred at 250 rpm. Upon addition of APTES (0.175 mL, 0.75 mmol), the mixture was stirred for 20 h at 85 °C. After centrifugation, the MSN were washed thoroughly with ethanol twice and acetone once in an ultrasonic bath for 30 min each. Finally, the particles were dried in vacuo at 50 °C overnight to yield “MSN-NH2”.

### Labelling of MSNs with a fluorescent dye

For in vivo imaging, the amino-functionalized particles (MSN-NH2) were fluorescently labeled with a Cy5-NHS dye. The particles were dispersed in 25 mM HEPES buffer (270 mg, 22 mg mL^−1^) at pH 7.2. Subsequently, a stock solution of Cy5-NHS in DMSO (7.49 µM, 2.16 mL, 16.179 µmol) was added, as well as additional EDC (76.6 mg, 401.072 µmol) and Sulfo-NHS (89.7 mg, 413.463 µmol) to compensate for potential hydrolysis-related loss of NHS ester. After agitation for 17 h on ice, the particles were separated via centrifugation and thoroughly washed with water once and ethanol ten times in an ultrasonic bath until complete absence of fluorescence in the washing media was observed. Finally, the particles were dried in vacuo at 60 °C overnight to yield fluorescently labeled “MSN-NH2-Cy5”.

For in vitro imaging, the amino-functionalized particles were fluorescently labeled with an ATTO647N dye. MSN-NH2 were dispersed in 25 mM HEPES buffer (270 mg, 10 mg mL^−1^) pH 7.2. Subsequently, 270 µl of ATTO647N-NHS in DMSO (1.555 µM, 0.420 µmol) and 208 µl of an aqueous stock solution of EDC (7.32 µM, 1.523 µmol) and NHS (15.4 µM, 3.203 µmol) were added. After agitation at RT for 1 h the particles were separated via centrifugation and thoroughly washed with water once, ethanol thrice and methanol twice in an ultrasonic bath until complete absence of fluorescence in the washing media was observed. Finally, the particles were dried in vacuo at 60 °C overnight to yield fluorescently labeled MSN-NH2.

The calcined MSN-c particles were fluorescently labeled with an ATTO647N dye via silane grafting as previously described by Beitzinger et al.^50^ Briefly, DMSO stock solutions of ATTO647N-NH2 (1.092 µM, 0.551 mL, 0.602 µmol), triethylamine (53.256 µM, 0.015 mL, 0.799 µmol) and 3-(triethoxysilyl) propyl isocyanate (29.916 µM, 0.027 mL, 0.796 µmol) were mixed in 30 mL dichloromethane in a dry Teflon tube under argon atmosphere. The mixture was stirred at 45 °C for 17 h. Subsequently, 20 mL of dichloromethane and a dispersion of dried (in vacuo, 100 °C, 24 h) MSN-c (150 mg, 15 mg mL^−1^) was added and the mixture was stirred at 45 °C for another 17 h. The solvent was removed with a rotary evaporator and the particles were thoroughly washed four times with ethanol and three times with methanol using an ultrasonic bath until complete absence of fluorescence in the washing media was observed. Finally, the particles were dried in vacuo at 60 °C overnight to yield fluorescently labeled MSN-c.

### Grafting of PEG onto MSNs

MeO-PEG_5k_-NHS (2.373 mM, 7.5 mL, 17.8 µmol) was dissolved in 25 mM HEPES buffer pH 7.2. To compensate for potential hydrolysis-related loss of NHS ester, EDC (17.3 mg, 90 µmol) and sulfo-NHS (39.1 mg, 180 µmol) were added, and the mixture was agitated for 35 min at RT. PEG-NHS solution (2.373 mM, 3 mL, 7.1 µmol) was mixed with a dispersion of fluorescently labeled MSN-NH2 (90 mg, 12.9 mg mL^−1^) and the reaction proceeded for another 3 h. Upon centrifugation, the particles were thoroughly washed with water once and ethanol thrice in an ultrasonic bath for 20 min each and dried in vacuo at 60 °C overnight to yield fluorescently labeled “MSN-PEG”. The amount of PEG grafted to the particles was determined by TGA. To correct for hydrolysis related mass losses, reference particles without the addition of PEG were analyzed as well.

### Grafting of HA onto MSNs

HA (6 mg mL^−1^, 10 mL) was dissolved in 25 mM HEPES buffer at pH 7.2 under agitation overnight. After the addition of EDC (192 mg, 1 mmol) and sulfo-NHS (325 mg, 1.5 mmol), the mixture was agitated for 40 min at RT. Activated HA solution (6 mg mL^−1^, 3 mL, 18 mg) was mixed with a dispersion of fluorescently labeled MSN-NH2 (90 mg, 12.9 mg mL^−1^) and the reaction proceeded for another 3 h. Upon centrifugation, the particles were thoroughly washed with water once and ethanol thrice in an ultrasonic bath for 20 min each and dried in vacuo at 60 °C overnight to yield fluorescently labeled “MSN-HA”. The amount of HA grafted to the particles was determined by TGA. To correct for hydrolysis related mass losses, reference particles without the addition of HA were analyzed as well.

### Fluorescence labelling of chitosan and adsorption onto MSNs

Chitosan was fluorescently labeled with an ATTO565 dye based on a procedure described by Huang et al.^51^. Briefly, an aqueous stock solution of chitosan in 0.1 M acetic acid (5 mg mL^−1^, 10 mL, 50 mg) was mixed with a stock solution of ATTO565-NHS in DMSO (1.412 µM, 0.5 mL, 0.706 µmol) in 10 mL methanol. To compensate for potential hydrolysis-related loss of NHS-dye ester, EDC (0.7 mg, 3.65 µmol) and NHS (0.8 mg, 6.95 µmol) were added, and the mixture was agitated at 40 °C for 4 h, and dialyzed in 4 L of distilled water for 40 h. The fluorescently labeled chitosan was precipitated with 0.1 M sodium hydroxide solution and separated via centrifugation at 4 °C. The precipitate was thoroughly washed six times with water until complete absence of fluorescence in the washing media was observed, and finally freeze-dried to yield “Chit-565”.

Functionalization of particles with ATTO565 labeled chitosan was achieved via adsorption of the polymer onto ATTO647N labeled MSN-c in 1 M sodium acetate buffer at pH 5. A stock solution of fluorescently labeled Chit-565 (4 mg mL^−1^, 0.45 mL, 1.8 mg) was mixed with a dispersion of fluorescently labeled MSN-c (30 mg, 5.4 mg mL^−1^) and the mixture was agitated for 2 h at RT. Upon centrifugation, the particles were thoroughly washed with water once, ethanol twice and methanol once until complete absence of fluorescence in the washing media was observed. The particles were dried in vacuo at 60 °C overnight to yield fluorescently labeled “MSN-Chit”. The amount of chitosan adsorbed onto the particles was determined by fluorescence intensity measurements of the adsorption supernatant.

### Characterization of silica nanoparticles

Thermogravimetric analysis (TGA) was performed at a heating rate of 10 °C min^−1^ in nitrogen/oxygen (70%/30%) atmosphere on a TG209 F1 Libra (Netzsch, Germany). Nitrogen sorption measurements were conducted on a Quadrasorb-1 (Quantachrome Instruments, Germany) at −196 °C after drying the particles in vacuo at 100 °C for 22 h. The specific surface areas were determined using the BET method. Pore diameters and pore volumes were calculated via equilibrium NLDFT kernel (silica, cylindrical pores) in a relative pressure range of p p_0_^–1^ = 0–0.9. The external surface areas were estimated by t-plot method in the relative pressure range of 0.62–0.78. Zeta potentials were measured three times with a Zetasizer NanoZS Zen3600 (Malvern Panalytical, Germany) in aqueous 1 mM KCl solution or 25 mM HEPES buffer (pH 7.4) and a particle concentration of 0.1 mg mL^−1^. Particle shape and size were examined with a Jeol 1200 (Jeol, Germany) transmission electron microscope (TEM) using a HT voltage of 120 kV and beam current of 65 µA. Electron micrographs of thin section were recorded after embedding the MSNs in Epon^®^ resin and cutting with a microtome. Fluorescence intensities of the labelled particles were measured with a Spark 10 M microplate reader (Tecan, Crailsheim, Germany).

### Dissolution experiments

Dissolution of MSNs were examined in fasted state intestinal fluid (FaSSiF) buffer composed of sodium taurocholate (3 mM), disodium maleate (19.12 mM), sodium chloride (68.6 mM) and lecithin (0.2 mM) adjusted with hydrochloric acid to a pH of 6.5^52^. The particles were dispersed in the buffer at a concentration of 1 mg mL^−1^ using a focused ultrasonic bath and diluted 1:1 resulting in a concentration of 500 µg mL^−1^ (normalized on rest mass at 600 °C). The dispersions were agitated for 14 h at 37 °C and centrifuged at 20,000 rpm for 15 min. Fluorescence intensities of supernatants and non-incubated dispersions were measured in 96 well plates. After centrifugation, the particles were briefly washed with acetone, centrifuged, re-dispersed in acetone, and finally pipetted onto TEM grids for electron microscopy.

### Cell cultures

Human colon adenocarcinoma cell line Caco-2 (Acc No. 86010202) and HT29-MTX-E12 (“HT29”) cells (Acc No. 12040401) were obtained from the European Collection of Authenticated Cell Cultures (ECACC, UK) and cultured in DMEM containing 20% FBS, 1% MEM non-essential amino acid solution, 1% l-Glutamine and 1% penicillin–streptomycin solution in a humidified atmosphere of 5% CO_2_ at 37 °C. Human lymphoma cell line Raji (Acc No: 85011429) was purchased from ECACC and grown in RPMI medium containing 10% FBS, 1% l-Glutamine and 1% penicillin–streptomycin solution in a humidified atmosphere of 5% CO_2_ at 37 °C.

### Evaluation of cellular toxicity

1.1 × 10^6^ Caco-2 cells were seeded in a 96-well plate for 48 h. The particles were dispersed in DMEM/10% FCS with a focused ultrasonic bath at different concentrations and added to the cells by diluting them 1:1, resulting in particle concentrations of 500 µg mL^−1^, 250 µg mL^−1^, 100 µg mL^−1^ and 50 µg mL^−1^ (normalized on silica rest mass at 600 °C), respectively. Then, cells were incubated with the respective components for either 6 h or 24 in serum containing medium for cytotoxicity measurements. After the defined time points, the medium was removed, and the cells were washed once with 100 µL PBS. 100 µL of fresh medium was added to each well and cell viability was measured using the CellTiter 96^®^ AQ_ueous_ One Solution Cell Proliferation Assay (MTS) according to the manufacturer’s instruction. In short, a volume of the MTS substrate equal to 10% of the volume of cell culture medium present in each well was added, cells were further incubated for 2 h at 37 °C and absorbance was measured at 490 nm using a TriStar^2^ LB 942 plate reader (Berthold Technologies, Bad Wildbad, Germany). Untreated medium only containing cells or cells treated with 20% DMSO served as negative and positive controls respectively. To visualize morphological changes, images before treatment and before addition of the MTS substrate were acquired using a LEICA DMi1 microscope connected to a LEICA MC170 HD camera (both Leica Microsystems GmbH, Wetzlar, Germany).

### In vitro co-culture model of the intestinal epithelium

For the development of the intestinal epithelium model, 5 × 10^4^ Caco-2 cells alone or Caco-2 and HT29 cells in a ratio of 7:3 were seeded on permeable PET membrane inserts. Cell culture medium was changed every second day. On day 14, 0.5 × 10^6^ Raji cells were added to the lower compartment. Cells were further cultivated for another 6 days while the Raji cells density was monitored and kept at 0.5 × 10^6^. For monitoring of the trans-epithelial electrical resistance (TEER), the medium was substituted with fresh cell culture medium and the electrode was inserted at a fitted depth and position. All TEER values were measured at RT using an EVOM3 Trans Epithelial Electrical Resistance meter (World precision instruments, Friedberg, Germany). Only co-cultures with comparable TEER values (> 300 Ω cm^−2^) were used for further experiments. 

For particle treatment, the MSNs were dispersed in xvivo15 medium with a focused ultrasonic bath at a concentration of 100 µg mL^−1^ (normalized on silica rest mass at 600 °C) and added to the cell cultures (n = 3) by diluting them 1:1 with xvivo15, resulting in a particle concentration of 50 µg mL^−1^. At distinct time points, the TEER values were measured as described before. For immunostaining, the particle containing media were removed and the cells were washed with PBS, fixed with PBS/4% PFA for 20 min, washed again with PBS and permeabilized with PBS/0.5% Triton for 15min. Afterwards, the cells were washed, incubated with 0.5 μL Alexa Fluor^TM^ 488-Phalloidin in 200 μL for 1 h, washed again and incubated with 1 µg mL^−1^ DAPI in PBS for 10 min. After another washing step, the membrane was cut out and molded onto the microscopy glass for confocal fluorescence microscopy.

CD44 overexpression was induced according to a previous report^53^. Briefly, co-cultures of Caco-2 and Raji cells were cultured as described before. At day 21, the cells were treated with IL-1β and LPS (50 ng mL^−1^ and 1000 ng mL^−1^) for 48 h. To confirm the overexpression of CD44, western blot analysis was conducted.

The cells were lysed in lysis buffer for 20 min on ice and centrifuged at 14,000 rpm for 20 min. The protein concentrations were determined using a BCA protein assay kit. The samples were mixed with protein sample loading buffer with 10% β-mercaptoethanol, heated at 95 °C for 5 min and 60–80 µg of protein was loaded onto 8–15% SDS–PAGE gels. The electrophoresed protein samples were blotted onto Immobilon-FL PVDF membranes using the antibodies anti-CD44 (ab254530) and anti-Actin (ab8227).

### Confocal laser scanning microscopy 

Confocal laser scanning microscopy was conducted with a Zeiss LSM 710 (Zeiss, Jena, Germany) equipped with an EC Plan-Neofluar 40x/1.30 oil objective and four lasers (405 nm, 488 nm, 561 nm, 633 nm). Images were acquired of randomly selected areas of the cell layers with a pixel scaling of 0.69 µm × 0.69 µm × 0.30 µm, an image size of 354.25 µm × 354.25 µm and a bit depth of 16 bit. All instrument settings were kept constant for image acquisition. Image analysis and post-processing was performed with ZenBlue software (version 3.7.97.0400). Black/white values were adjusted for each channel (nuclei: 1400/44,000, actin: 2000/50,000, MSN: 2800/65,500, chitosan: 2800/65,500) and were kept constant for all acquired images. Maximal intensity projections and 3D rendered projections of on-top views were created using the software-specific tools.

### Oral administration of MSNs in vivo

All animal-related experimental procedures and protocols in this study were approved by the Animal Ethics Committee of the University of Queensland, Brisbane, Australia and conducted according to National Health and Medical Research Council guidelines (AEC approval number: MRI-UQ/TRI/058/17), and are in accordance with ARRIVE guidelines. 6 weeks old, male C57BL/6J mice (n = 36) were randomized in groups of three individuals and held under standard laboratory conditions and with a 12 h light/dark cycle. After 7 days of settling, the animals were re-caged and their initial body weight was determined. For one half of the population water supply was replaced by an aqueous 1.5% dextran sulfate sodium solution (DSS) and the diet was changed to a fluorescence-free diet (Teklad Global Diet 2019S). The body weight and health score (diarrhea, rectal bleeding) of the numbered animals were daily monitored for another 7 days during which access to water was provided over the whole course. 12 h prior to administration the mice were set on a fasted state to minimize influence of food matter and feces and DSS containing water was replaced by regular water. The particles were dispersed in PBS at a concentration of 20 mg mL^−1^ using an ultrasonic probe and administered via careful oral gavage to the stomach using gavage needles. The single particle dose of 100 mg kg^−1^ was normalized on the current body weight, and 110 µL of PBS served as negative control. For each treatment/duration/health state combination three animals were used (n = 3). Sample blinding was not considered in this study as dosage determination and administration was dependent on health status and body weight of each individual numbered animal. The animals were placed back in their cages and provided with fresh water. After 6 h and 14 h, the mice were sacrificed, and the organs and GITs were immediately retrieved. All handling of live animals and organ excisions were performed by the same two investigators to ensure consistency in animal handling and reduction of stress. Particle gavage was carried out by a single researcher to ensure standardized experimental procedure. Fluorescence intensities were immediately measured using the in vivo imaging system IVIS (PerkinElmer, Waltham, USA) by placing the organs on black paper sheets. Imaging was conducted at an excitation wavelength of 570 nm (autofluorescence correction) and 640 nm and an emission wavelength of 670 nm. Image analysis was conducted with Living Image 4.8.0 (PerkinElmer, Waltham, USA). Background fluorescence subtraction for all images was conducted in order to exclude light reflection off stage. Regions of interest (ROI) were defined for each organ and the total radiant efficiencies were measured. In order to account for tissue related autofluorescence an organ specific correction factor (k_HC/DC_ = ROI_640nm_/ROI_570nm_) was calculated for healthy (HC) and DSS (DC) control animals (i.e. animals treated with PBS only) respectively for each image sequence recorded and the treated samples were corrected with the respective correction factors (fluorescence intensity = ROI_640nm_ − (k_HC/DC_ · ROI_570nm_)).

### Data analysis

The reported data are either individual values or mean values ± standard deviation (SD). The sample size (n) is specified in the respective figure legends and table captions. Particle size was determined using Zeiss ZenBlue 3.7 and a total number of 40 particles or higher was measured.

## Results

### Silica nanoparticle characterization

**Figure 1:**
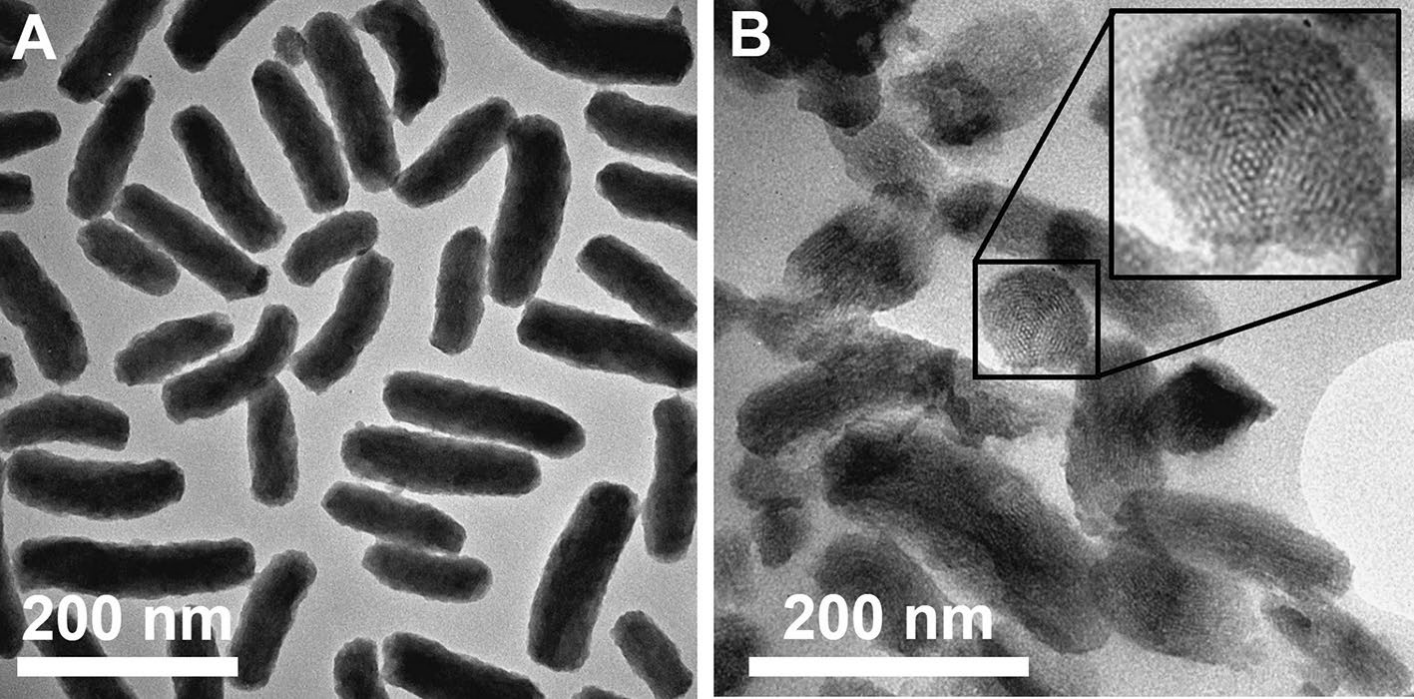
Transmission electron microscopy images of MSNs. (**A**) TEM image of representative basic calcined MSN-c particles. (**B**) TEM image of thin sections display the porous structure of MSNs.

Rod-shaped mesoporous silica nanoparticles with a length of 149 ± 46 nm and a width of 54 ± 7 nm resulting in an aspect ratio of 2.7 were synthesized. Exemplary transmission electron micrographs (TEM) of the particles and ultra-thin sections are shown in Fig. 1. The particles exhibited a 2D hexagonal, MCM-41-like pore system which was aligned longitudinal to the particle’s axis. CTAB used as the porogen was removed by calcination at 550 °C. The resulting all-silica particles (“MSN-c”) exhibited a specific surface area of 920 m^2^ g^−1^ as well as a pore size and pore volume of 3.2 nm and 0.58 cm^3^ g^−1^. The external surface area was 157 m^2^ g^−1^ as calculated by the t-plot method. Nitrogen sorption isotherms and pore size distribution obtained by NLDFT calculations can be seen in Supplementary Fig. S1. The calcined particles were further covalently post-functionalized with an amino-silane (“MSN-NH_2_”), which led to a change in the zeta potential from a negative (−32 mV) to a positive (+ 15 mV) as measured in HEPES buffer at pH  7.2 (Table 1). Further functionalization through covalent attachment of a Cy5 fluorophore via EDC/NHS coupling (“MSN-NH2_Cy5”) shifted the zeta potential back to a negative value of −26 mV due to the dominating extent of free silanol groups compared to decreasing amounts of primary amino groups after the dye attachment. The presence of free amino-groups after dye attachment was evident by a zeta-potential of + 28 mV as measured in non-buffered 1 mM KCl (pH  5.7) (Supplementary Figure S2). The free amino groups were utilized to further covalently functionalize the particles with HA (“MSN-HA”) and PEG (“MSN-PEG”). The degree of functionalization was measured via thermogravimetric analysis (TGA) (Supplementary Fig. S3), where the mass loss in the temperature range of 150°C to 700°C of each particle was normalized on the particle weight at 150 °C. Additionally, the mass loss was corrected for silica condensation-connected mass loss, by use of a reference sample which was treated under the same synthesis conditions, but without the addition of polymer. The quantity of HA and PEG attached to the MSNs were rather low (3.7 wt% and 4.7 wt%), which may be caused by partial obstruction of the external amino functions by the fluorophore and/or diffusion limitation of polymers into the porous space, where the latter would result in predominant functionalization of the outer particle surface. Nevertheless, the amount of grafted polymer was sufficient to further shift the zeta potential of MSN-HA towards more negative values as compared to the fluorescently labeled particles. Contrary to that, no change of absolute zeta potential was observed upon PEGylation.

**Table 1:** Zeta-potentials measured in 25 mM HEPES buffer (pH 7.4) and functionalization degrees measured via TGA of synthesized MSNs (zeta potentials: mean ± SD, n = 3).

	Functionalization degree (wt.%)	Functionalization degree (µmol g^−1^ SiO_2_)	Zeta potential (mV)
MSN-c	–	–	− 32 ± 5
MSN-NH2	9.1	56.29	+ 15 ± 3
MSN-NH2_Cy5	–	–	− 26 ± 5
MSN-PEG	4.7	11.68	− 22 ± 4
MSN-HA	3.7	0.03	− 34 ± 4

### Mesoporous silica nanoparticles do not dissolve under simulated GIT conditions

**Figure 2:**
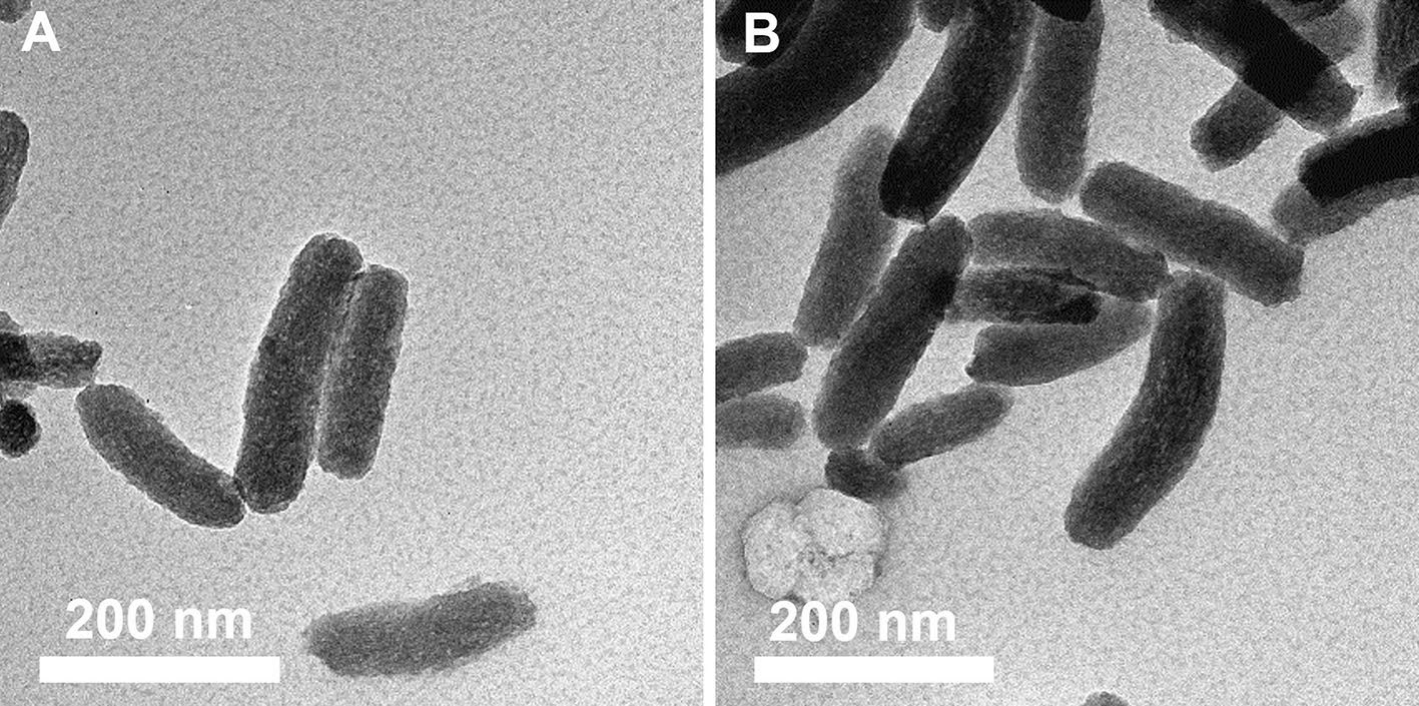
Transmission electron microscopy of (**A**) MSN-HA and (**B**) MSN-PEG after dissolution experiments. The particles were incubated in FaSSIF buffer (pH 6.5) for 14 h under agitation at 37 °C, and at a rest mass-normalized silica concentration of 500 µg mL^−1^.

Silica nanoparticles are well-known for their ability to dissolve in aqueous media which is an important key parameter for biodegradability and biocompatibility. Mesoporous all-silica nanoparticles have been reported to completely dissolve in simulated body fluid at neutral pH within hours^54^. However, therapeutic platforms intended for oral administration of drugs need to be intact long enough to ensure the delivery of their cargo to the site of therapeutic action, e.g. the colonic wall. To assess whether our particles could withstand premature dissolution under dilute conditions, the MSNs were incubated under agitation in FaSSIF buffer (pH 6.5) at 37 °C, and a rest mass-normalized silica concentration of 500 µg mL^−1^ for 14 h. After incubation, the particles were recovered by centrifugation, and their structural integrity was examined via TEM (Fig. 2). The functionalized MSNs showed only minor signs of dissolution in the aqueous buffer, and the pore structure was still intact. Also, no alteration of particle length or width was observed. Thus, it can be concluded that structural integrity is preserved over time courses relevant for oral administration of MSNs. Moreover, fluorescence measurements of the dissolution supernatants revealed that around 85% of the dye were still attached to MSN-HA and MSN-PEG respectively after incubation (Supplementary Fig. S4) confirming that the particle’s fluorescent label and polymeric functionalization can withstand this treatment. However, the conditions simulated here are of course not fully mimicking the conditions the particles would encounter under in vivo conditions, where the composition of the intestinal fluid is much more complex and is comprised of a wide variety of constituents, e.g. proteins, fatty acids or food particles. In general, adsorption of biological compounds is known to decrease the dissolution kinetics of mesoporous silica^55–57^. Recently, Iqbal et al*.*^58^ demonstrated that elongated mesoporous silica particles with a length of 1 µm were not degraded after complete passage through the whole GIT in mice as well as in human trials and that the pore structure remained unaffected even under the strongly varying pH values present in the GIT. In the present study, the particles were orally administered in mice as a single particle dose of 100 mg kg^−1^. Considering the body weight and overall gastrointestinal volume of average mice to be approximately 20 mg and 1.34 mL respectively^59^, this would result in a mean concentration along the GIT of 1.5 mg mL^−1^. This is much higher than the reported silica solubility limit of roughly 120 µg mL^−1^ under low salt conditions^60^, and the particle concentrations used in our in vitro dissolution experiments, which were conducted under even more dilute conditions in compliance with a more fluid gastro intestinal content as it is typical for diseases affecting the GIT (e.g. IBD). This gives further support that the particles used in this study are stable against dissolution under the in vivo conditions.

### Intestinal distribution of MSNs depends on functionalization type

**Figure 3:**
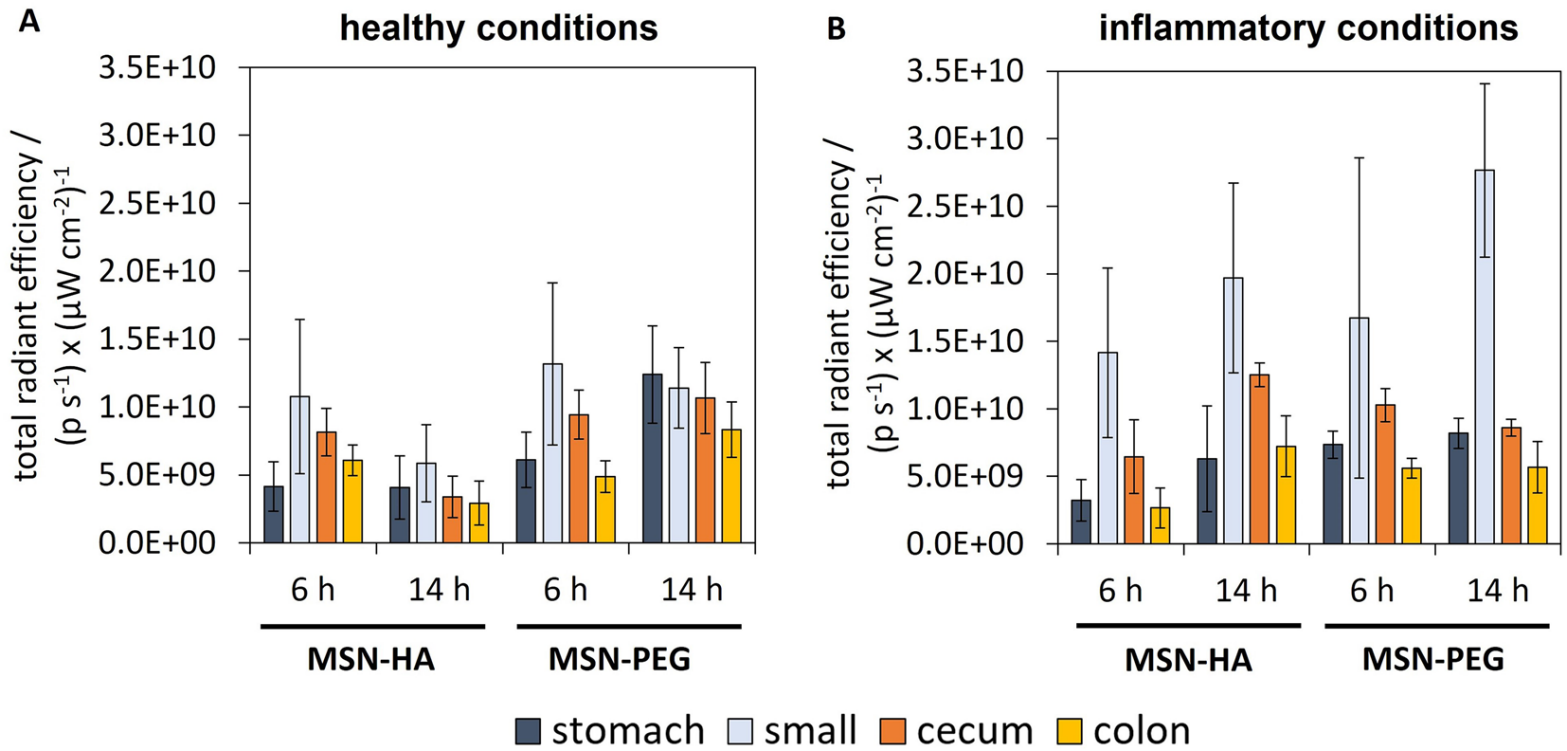
Gastro-intestinal distribution of fluorescently labeled MSNs. GITs were excised from (**A**) healthy mice and (**B**) mice with DSS-induced inflammation 6 h and 14 h after oral gavage of a single dose of 100 mg kg^−1^. Fluorescence intensities were measured via IVIS with an excitation/emission wavelength of 640/670 nm and were corrected for tissue-related autofluorescence based on control animals. (mean ± SD, n = 3).

To investigate the local distribution within the GIT depending on the state of inflammation, a well-established in vivo mouse model to mimic the alteration of the mucosal barrier was used^41,42,61,62^. Erosion of the mucosal barrier is one of the symptoms of inflammatory diseases like ulcerative colitis. DSS containing water was orally administered to mice over a course of seven days, and the state of inflammation was indicated by the development of diarrhea and bloody stool consistency. In general, DSS-related effects are limited to the lower GIT (colon and cecum) epithelium resulting in reproducible alteration of the mucosal barrier, decreasing epithelial structures, as well as formation of inflamed lesions caused by recruitment of immune cells such as macrophages and granulocytes. Fluorescently labeled MSN-HA and MSN-PEG were dispersed in PBS and orally administered to healthy and DSS-treated mice via gavage as a single dose of 100 mg kg^−1^, which corresponded to approximately 2 mg per mouse. Fluorescence intensities of both particle types were similar as shown in Supplementary Fig. S5. After 6 h and 14 h mice were sacrificed, and GITs as well as organs were excised. Fluorescence intensities were measured using an in vivo imaging system (IVIS) with an excitation/emission wavelength of 640/670 nm. Representative fluorescence images of GITs are shown in Supplementary Fig. S6. Tissue-related autofluorescence was corrected for by recording additional images with an excitation/emission wavelength of 570/670 nm for control animals that had not been treated with MSNs (see experimental for details). Autofluorescence-corrected intensities of stomach, small intestine, cecum and colon 6 h and 14 h after gavage are shown in Fig. 3.

At 6 h post-administration, particles were already present in the lower GIT regions (cecum and colon) irrespective of the health state and particle surface chemistry. This finding is in line with reported transit times of 6–8 h in healthy mice^63^. Thus, the 6 h time point was used as a reference for further analysis of different particle behavior over time within one model. However, a direct cross-comparison of particle retention at fixed time-points between models is difficult due to increased gastro-intestinal transit times during the acute phase of DSS-induced colitis^64,65^. Thus, we based our discussion on comparison of results obtained for a given mouse model. 

14 h after gavage of MSN-PEG to healthy animals, the intensity in the small intestine was very similar to that measured after 6 h. This would indicate a steady particle influx from the stomach. The role of the stomach as long-lasting particle reservoir is in line with results reported in a recent study, where solid lipid nanoparticles were found in the stomach even 12 h post-gavage^66^. However, the MSN-PEG particles were more abundant in the lower GIT at 14 h as compared to 6 h post-gavage, indicating some level of particle retention in this region. Interestingly, the exact opposite was the case for MSN-HA in healthy mice, for which particle-related fluorescence was decreased by a factor of around 2 for the small intestine, the cecum and the colon between the 6 h and the 14 h time-point, highlighting the GIT clearance of these MSNs. In mice with DSS-induced inflammation, the amount of MSN-PEG present in the GIT remained unchanged over time, again indicating particle influx from the stomach. Intriguingly, a completely different picture can be drawn for MSN-HA in DSS-treated mice. A strong increase in particle fluorescence over time was observed for the cecum and the colon by a factor of about 2 and 3 respectively.

**Figure 4:**
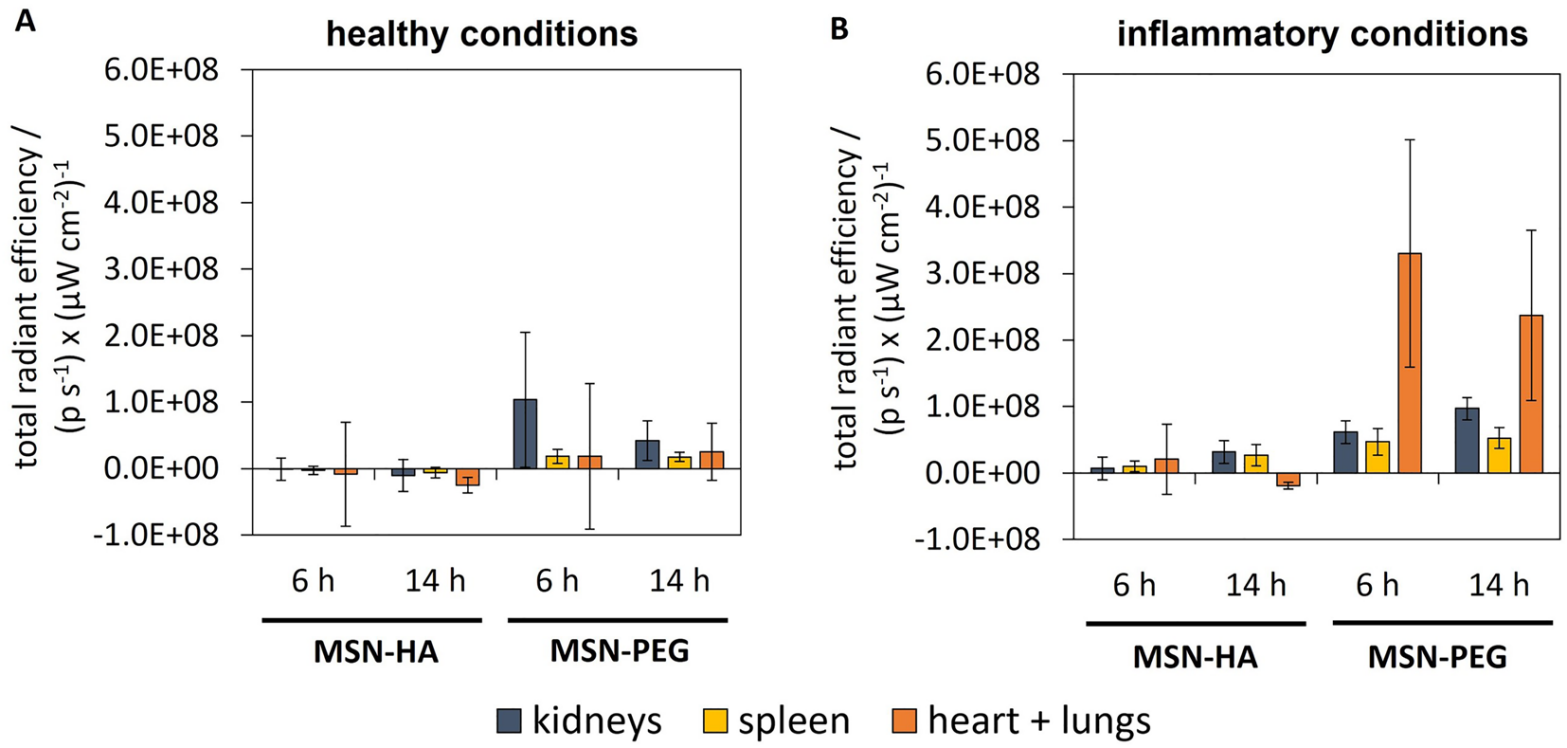
Bio-distribution of fluorescently labeled MSNs in organs. The organs were excised from (**A**) healthy mice and (**B**) mice with DSS-induced inflammation of the GIT 6h and 14h after oral gavage of a single dose of 100 mg kg^−1^. Fluorescence intensities were measured via IVIS with an excitation wavelength of 640 nm and an emission wavelength of 670 nm. Auto-fluorescence correction was conducted as stated in the experimental section. (mean ± SD, n = 3).

In addition to the particle distribution in the GIT, we examined if the particles could permeate through the epithelium and be absorbed into systemic circulation. Therefore, the internal organs were excised 6 h and 14 h post-gavage and analyzed for particle-related fluorescence. Exemplary fluorescence images of organs recorded via IVIS can be seen in Supplementary Fig. S7. Figure 4 shows autofluorescence-corrected intensities measured for the excised organs 6 h and 14 h post-gavage. Tissue-related autofluorescence levels were high, especially for large organs rich in blood like the liver, which produced unreliable data after correction for autofluorescence based on the control animals as described in the experimental section. This data was therefore omitted in the analysis. At 6 h, no MSN-HA particles were detected in the organs irrespective of the health status of mice. MSN-PEG content in organs of healthy animals was neglectable and high mouse-to-mouse variation impeded detailed data analysis. However, fluorescence levels of MSN-PEG measured after 6 h in DSS-treated mice were evident and notably increased by a factor of 18 and 2.5 for the heart/lungs and the spleen, respectively. In a previous study by Kramer et al.^67^, PEGylated MSNs were found to have a pro-longed blood retention time when injected intravenously, and accumulation thereof preferentially happened in the organs of the immune system, mainly in the spleen. In the same study, high particle contents were also found in blood-rich organs like the heart/lungs due to high MSN concentrations in the blood, which could be allegedly misinterpreted as actual accumulation. Presumably, this was also the case in the present study, supported by the fact that MSN-PEG contents in diseased animals changed from 6 to 14 h. While the intensity in the heart/lungs decreased by a factor of 0.7, fluorescence levels in the kidneys and the spleen were further increased by a factor of 1.6 and 1.1, respectively. This indicates the beginning of particle clearance from the blood circulation. Weak fluorescence levels of MSN-HA were only found in the kidneys and the spleen of DSS-treated mice 14 h post-gavage, in contrast to the fit animals. In healthy mice, MSN-PEG concentrations were low indicating that PEGylated particles were able to permeate through the healthy epithelium to some minor extent. Nevertheless, particle absorption in the GIT was clearly influenced by the treatment with DSS suggesting that inflammation and disruption of the epithelial barrier can promote particle uptake into the system. However, overall organ-related intensities were minor compared to intensities measured in the GIT, indicating that the vast majority of particles remained in the GIT and were not absorbed into systemic circulation. This indicates that rod-like MSNs would be promising candidates for local, prolonged drug delivery within the GIT.

### Presence of mucus influences accumulation of MSNs on epithelial cell models

**Figure 5:**
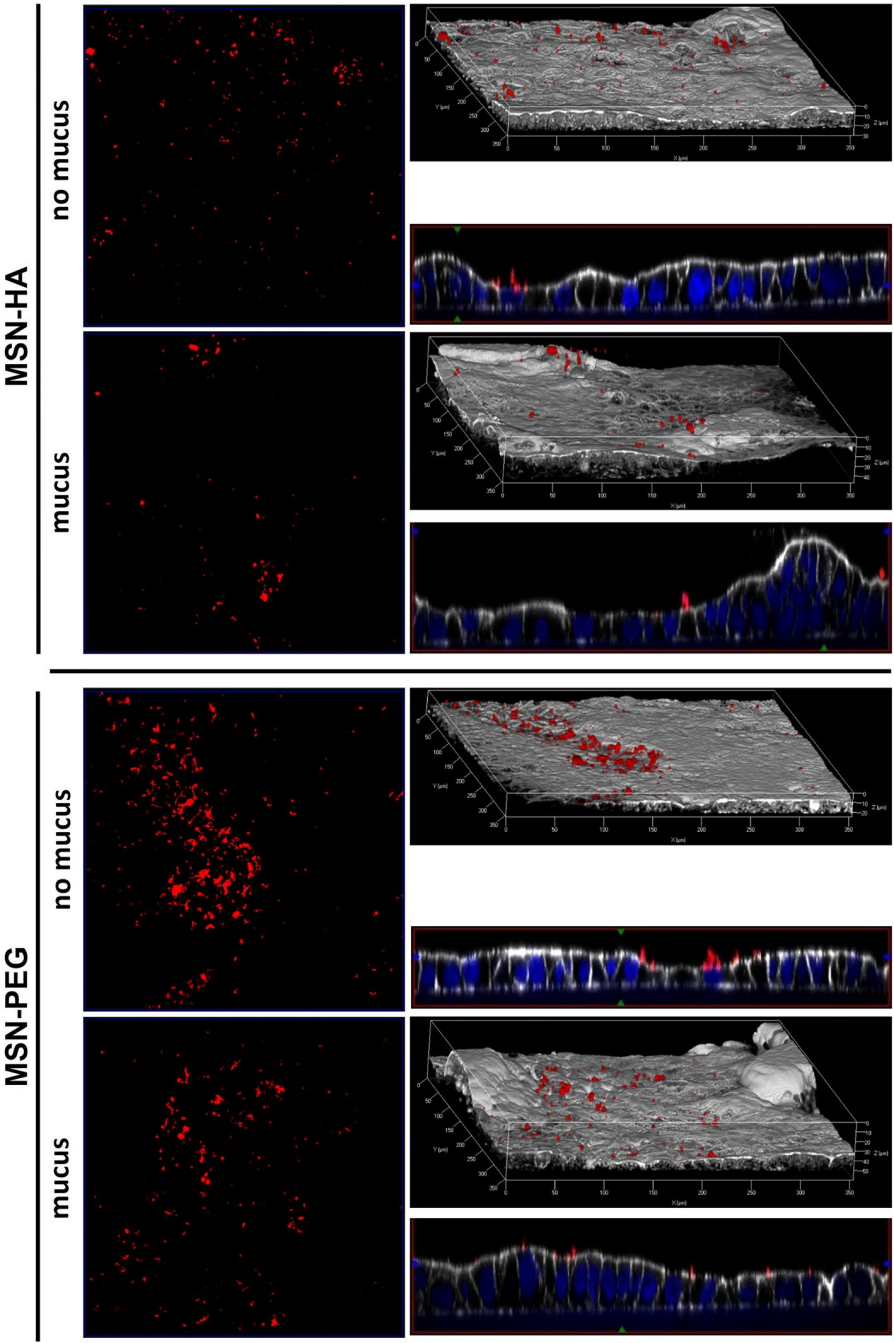
Adherence of MSN-PEG and MSN-HA to epithelial cell models. Representative confocal laser scanning microscopy images of random areas of Caco-2/Raji (“no mucus”) or Caco-2/HT28/Raji (“mucus”) co-culture cell models after incubation with MSN-PEG or MSN-HA at a silica concentration of 50 µg mL^−1^ for 14 h at 37 °C. Left column: maximal intensity projection of on-top views of the epithelial model. Right column: channel overlay of 3D rendered projections and cross-sectional Y/Z side views of channel overlays. MSNs (red), cell membrane (white), nuclei (blue). (n = 3).

To further investigate the influence of particle surface chemistry on trans-epithelial localization and resorption, epithelial cell models with and without a mucus layer were used. Cell co-cultures comprised of Caco-2/Raji (“no mucus”) or mucin producing Caco-2/Raji/HT29 (“mucus”) were cultivated on permeable membranes. Fluorescently labeled MSNs were added to the apical chamber at a silica concentration of 50 µg mL^−1^ and incubated for different periods of time. After several washing steps, the cells were fixed, fluorescently stained and examined via laser scanning confocal microscopy. On-top views of maximum projections of the samples as well as rendered 3D projections and side views after incubation for 14 h are shown in Fig. 5 and additional time points can be found in Supplementary Fig. S8.

Both negatively charged MSN-HA and MSN-PEG were present on cells without mucus, and the increase of particle fluorescence intensity over time was more pronounced for MSN-PEG. Overall intensities for MSN-PEG were higher as compared to MSN-HA. However, we cannot fully exclude that sedimentation effects may also played a role for this observation. Thus, in the following we compared results obtained for mucus vs. no mucus conditions. Both studied MSNs were located on top of the epithelium and close to the cell membranes. In all cases, neither cellular uptake nor permeation of the particles into or through the cell layer were observed. Impermeability of Caco-2-based epithelial models for silica nanoparticles has been previously reported for particles with an even smaller size of 50 nm^5^. With regards to reported CD44 overexpression in inflamed intestinal regions, which can act as a potential target for HA-based drug transporters^47,68^, we asked ourselves if modelling these conditions in vitro would have a pronouncing effect on particle uptake. In connection to this, self-assembling HA-based nanoparticles were reported to be internalized into CD44 expressing cells^53^. However, CD44 overexpression induced by treatment with pro-inflammatory IL-1β and LPS of Caco-2 mono-cultures had no effect on particle internalization or permeation across the model epitheliums (Supplementary Fig. S9), whether for MSN-PEG nor MSN-HA. This is advantageous if extracellular release of therapeutic drugs at the site of inflammation is desired.

**Figure 6:**
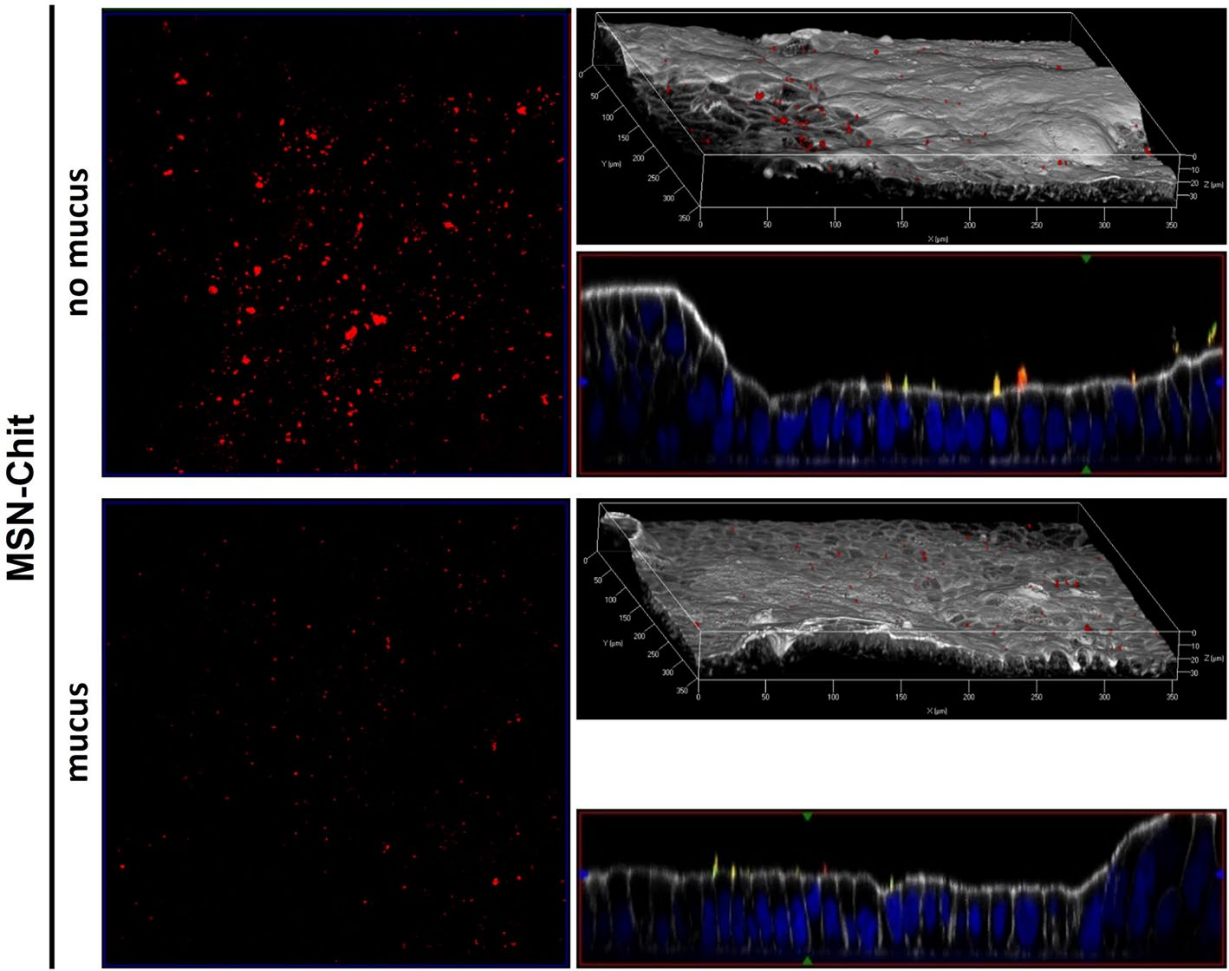
Adherence of MSN-Chit to epithelial cell models. Representative confocal laser scanning microscopy images of random areas of Caco-2/Raji (“no mucus”) or Caco-2/HT28/Raji (“mucus”) co-culture cell models after incubation with chitosan-functionalized MSNs at 50 µg mL^−1^ for 14 h at 37 °C. Left column: maximal intensity projection of on-top views of the epithelial model. Right column: 3D rendered projections and cross-sectional Y/Z side views of channel overlays. MSNs (red), Chitosan (green), cell membrane (white), nuclei (blue) (n = 3).

However, also no particle internalization was observed in the mucin producing epithelial cell model irrespective of surface functionality, and even less MSN-HA was located on the epithelium as compared to the cell model without mucus. In contrast, the abundance of MSN-PEG was still high and less affected by the presence of mucus. We hypothesized that this was due to MSN-HA particles being unable to penetrate through the mucin layer and eventually accumulate, while MSN-PEG could penetrate the mucus. Hu et al.^66^ reported that the adherence of solid lipid nanoparticles on Caco-2 and Caco-2/HT29 cell co-cultures was strongly compromised by the presence of the mucus layer because of their inability to penetrate through the layer, which is why they were easily washed away during further staining procedures. We speculated that this was also the case for MSN-HA in the present study. To verify this hypothesis, fluorescently labeled MSNs having surface silanol groups (zeta potential = − 35 mV) and comparable in size to the other MSNs studied, were further functionalized with chitosan, a polymer well-known for its mucus-impermeable properties, through adsorption from aqueous solution. The chitosan loading degree was 5.3 wt%. These particles are denoted MSN-Chit in the following. The pK_a_-value of protonated chitosan is around 6.1–6.3^69^, which is why the MSN-Chit exhibited a negative zeta potential of −27 mV at physiological pH. Analogous to MSN-HA and MSN-PEG, the MSN-Chit particles showed no signs of dissolution or premature dye detachment in FaSSIF buffer, nor cytotoxicity (Supplementary Figs. S4, S10, S11). In order to study potential detachment of chitosan under physiological conditions, the chitosan itself was fluorescently labeled. The fluorescence originating from chitosan co-localized with fluorescence originating from the MSN, giving proof for the stability of the adsorptive polymer functionalization. The in vitro results obtained for MSN-Chit are shown in Fig. 6. No internalization of MSN-Chit was observed, and the particles were abundant on the mucus-lacking cell layer. However, on mucin-producing cells, the particles exhibited a similar behavior to that of MSN-HA. Almost no chitosan-functionalized particles were observed in this case, emphasizing the obstructing effect mucins exercise on diffusion and accumulation of such particles.

Overall, the in vitro results fit well to the observed GIT clearance of MSN-HA in the in vivo experiments. The intact mucus layer represents a diffusion barrier for HA-functionalized particles leading them to stay localized more towards the center of the lumen, and to be readily transported off-site along with intestinal flux. On the other side, diffusion limitation does not apply to MSN-PEG to the same extent, causing pronounced permeation towards the intestinal wall, eventually leading to slower clearance in healthy animals alongside with the possibility of (low) particle resorption. However, the comparison with the in vivo results also shows the limitation of cell-based in vitro models when it comes to modelling of diseases causing complex tissue alterations. No internalization into or permeation through the cell layers were observed, in contrast to notable increases in particle content in organs for MSN-PEG in mice under inflammatory conditions.

### The epithelium integrity was retained upon exposure to MSNs in vitro

**Figure 7:**
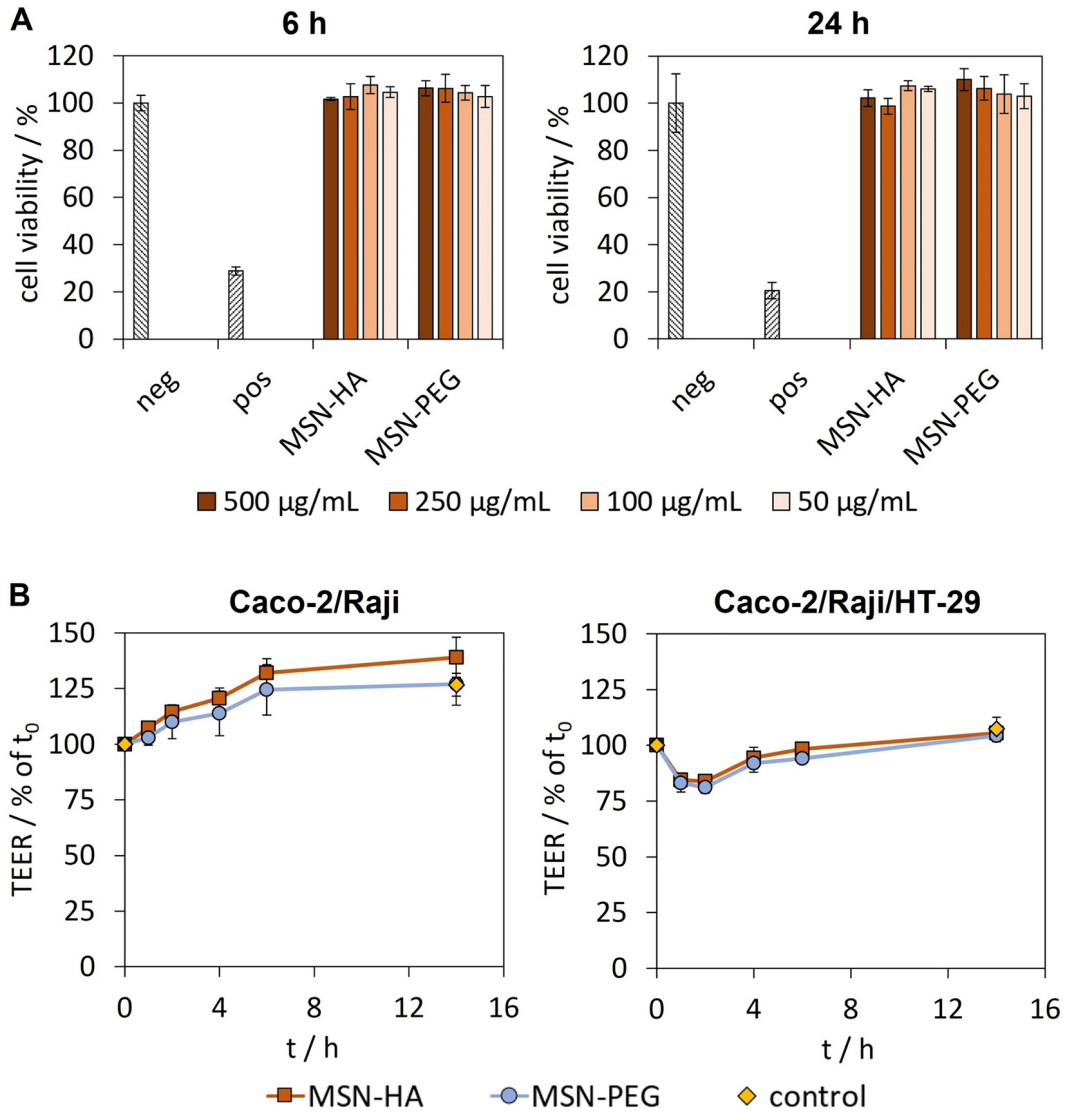
Toxicological evaluation of MSN-HA and MSN-PEG. (**A**) Cell viability of Caco-2 cells after incubation with varying particle concentrations after 6 h and 14 h as well as DMSO treated cells (pos) was measured with MTS-assay and was normalized on cells without treatment (neg). (**B**) Measurement of the TEER of Caco-2/Raji (left) and Caco-2/Raji/HT29 (right) epithelial models upon incubation with MSN-PEG or MSN-HA at 50 µg mL^−1^. TEER values were measured at distinct time points and calculated as percentage of TEER before the addition of the particles (mean ± SD, n = 3).

To exclude toxicity or epithelial damage caused by the administration of MSNs, viability of the colonic cell line Caco-2 was assessed via MTS assay after exposure to MSN-HA and MSN-PEG for 6 h and 24 h at varying particle concentrations. Figure 7A shows that no signs of cytotoxicity were observed at both incubation times and high particle concentrations of up to 500 µg mL^−1^, i.e. values exceeding those typically used for in vitro toxicity studies. Furthermore, cell morphology was not affected by incubation with the different MSNs as demonstrated by light microscopy images (Supplementary Fig. S12). The influence of therapeutics on the epithelial integrity as well as their accumulation and/or permeation through the epithelium are important key parameters to consider when designing promising therapy platforms intended for oral administration. Co-culture cell models comprised of the three different cell lines Caco-2, Raji and HT-29 can be utilized to draw conclusions about epithelial integrity and permeability to drugs or drug carriers by measuring the trans-epithelial electrical resistance (TEER) between the apical and basal side of the cellular layer^36,37^. MSNs were incubated on epithelial models comprised of Caco-2/Raji cells as well as cell cultures additionally including mucin producing HT-29 cells for up to 14 h at a silica concentration of 50 µg mL^−1^. The TEER were measured at distinct times and were normalized on the TEER values measured for each sample before the beginning of the incubation period. Figure 7B shows the particle-related effects on the TEER upon addition of MSNs. In the case of MSN-HA, Caco-2 TEER values were continuously increasing and reached a level after 14 h which was 139% higher than the original resistance levels. Rising TEER levels were also observed for control cultures without particles as well as for samples including MSN-PEG in the same extent (126% of t_0_), indicating that proliferation and consolidation of the Caco-2 epithelium continued. However, in the case of Caco-2/Raji/HT29 co-cultures, no continued condensing of the cellular layer was observed, but rather a decrease in TEER values to about 80% within 1 h upon addition of both particle types. Similar observations were previously reported in a study by Lamson et al*.*^5^ for the incubation of Caco-2 monolayers with silica nanoparticles at a concentration of 2 mg mL^−1^, and was shown to be inversely correlated with particle size, as particles with a diameter of 20 nm and 200 nm caused a drop in TEER to about 10% and 70% of initial values respectively within the first 3 h of incubation. A recent study by Cao et al.^6^ reported a decrease in TEER of Caco-2 mono-cultures and Caco-2/HT-29 co-cultures upon incubation with virus-like silica nanoparticles resulting in increased permeability of macromolecular therapeutics in vitro and in vivo. These findings were ascribed to a temporal and reversible opening of inter-cellular tight junctions, which could eventually be utilized to increase permeability of drugs through the epithelial layer. In the same studies, particle concentration dependency of TEER development was reported, and this may be the reason why no TEER drop was observed for Caco-2/Raji cell cultures in our experiments conducted at much lower particle concentrations. Compared to Caco-2 mono-cultures, permeability values for Caco-2/HT-29/Raji co-cultures are reported to be higher due to larger tight junctions^70^, rendering them more prone to particle related changes in TEER values even at low concentrations. Nevertheless, the TEER values of Caco-2/Raji/HT-29 co-cultures recovered to pre-treatment levels within the next 5 h, and finally reached a level identical to the control samples after 14 h of incubation. Overall, it can be concluded that the MSNs were non-toxic over a wide concentration range, and that there were no negative effects on the integrity of complex epithelial models in vitro.

## Discussion

Major challenges of oral drug delivery, *e.g.* decreased bioavailability, are supposed to be overcome by the development of delivery platforms. This would be especially the case for diseases altering the gastro-intestinal physiology and environment. To fulfill these high promises, long residence times in the GIT are a pre-requisite. Thus, this work aimed for the in-depth analysis of the intestinal and organ-specific biodistribution and retention of orally administered MSNs to mice with healthy and inflamed GIT over time. Size and shape of the particles were rationally chosen based on findings reported in literature and further expanded by the influence of surface functionality on the particle distribution in organs and GIT depending on the health status of the animals. Moreover, the obtained results from in vivo experiments were connected to differences in particle adhesion in vitro on cell models in the presence and absence of mucus.

In the lower GIT of healthy mice, increasing fluorescence intensities over time were detected in the case of MSN-PEG, whereas fluorescence intensities of MSN-HA decreased, indicating slower clearance of the PEGylated particles. Additionally, in our co-culture cell models adherence of MSN-PEG has been shown to be less affected by the presence of mucus. On the other hand, MSN-HA were less abundant on mucus-secreting cell layers as compared to the cell cultures without mucus, which was also the case for MSN-Chit. This implicates, that the different bio-distributions in vivo were based on alteration of mucus-penetrability by the polymeric functionalization leading to different luminal localization and excretion rates. Maisel et al*.*^24^ reported pronounced deposition of mucus-penetrating polystyrene particles at the intestinal wall via analysis of intestinal cross-sections. In contrast, their mucus-adhesive analogues were localized within the intestinal lumen unable to penetrate through the mucus towards the epithelium, resulting in transportation off-site along with the intestinal flux. However, no statements about differences regarding retention and resorption of such particles were made. Additionally, it was previously demonstrated that PEGylation of PLGA particles can inhibit mucin binding followed by increased particle mobility, as well as coverage on mucosal tissues, and effective penetration close towards the epithelium in vaginal and colorectal tissues in vivo^25,71^. These effects were reported to be dependent on the surface density of the PEG chains which were in the range of 0.07 to 0.1 chains/nm^−2^, i.e. values comparable to the MSN-PEG used in this study (0.05 chains/nm^−2^). In DSS-treated mice with damaged mucosal barriers a completely different trend was observed for the HA-functionalized particles as compared to MSN-PEG. Intensities in the cecum and the colon strongly increased over time, indicating high affinity towards the disrupted mucosa and potential accumulation of MSN-HA in the inflamed tissue. This finding fits well to a previous study where complex hierarchically structured 35 µm vehicles (AP@PSi-HA@HPMCAS) based on HA-functionalized silicon nanoparticles showed greater retention (but no tissue resorption) in mice with DSS-induced colitis as compared to healthy mice 2 h and 5 h post-administration, a result which was mainly ascribed to the negative particle charge^48^. Furthermore, it is reported that positively charged proteins such as transferrin or eosinophil are abundant on the surface of damaged epithelia which could potentially offer increased affinity of drug delivery platforms with a negative surface charge^44,45^. In another study, rectally administered HA-based hydrogels (without particles) showed increased affinity towards inflammatory regions of the GIT induced by TNBS treatment^72^. However, electrostatic interaction of such proteins is not expected to account for anionic PEGylated MSNs due to the well-known inhibiting effect PEG exerts on protein adsorption.

In general, resorption of bare silica nanoparticles by healthy intestinal tissue is reported to result in accumulation in the organs of the RES, where rod-shaped particles remained longer in the systemic circulation than spherical particles^16,17^. This stands in contrast to a study demonstrating increased drug permeability through opening of tight junctions by incubation with 50 nm spherical silica particles in vitro, but where no intestinal particle resorption was observed in vivo^5^. In the present study, mucosal inflammation and erosion in mice treated with DSS resulted in steady MSN-PEG amounts in the GIT, but the amounts in organs like kidneys, spleen and heart/lungs were increased. PEGylation has been reported as a means to increase the translocation and infiltration of nanoparticles on/into inflamed mucosal biopsies of IBD patients^29^, which explains their pronounced resorption into the system in our case and thus steady fluorescence intensities in the GIT. Additionally, colonic permeability in general has been demonstrated to be increased in DSS-induced colitis due to alteration of the tight junction protein ZO-1^73,74^.

Cell-based epithelial models (mono- and co-cultures) are a widely used means for toxicity and drug permeability studies^5,6,70,75,76^, and are also utilized to examine the epithelial transport of drug delivery platforms. Schimpel et al.^37^ compared the uptake of 50 and 200 nm polystyrene particles on Caco-2 mono-cultures as well as on mucus producing co-cultures. Internalization of particles was observed for Caco-2 cell layers. However, their uptake was strongly reduced by the presence of mucus which led to immobilization of the particles. Integration of M-cells into the epithelium again promoted internalization in all cases, and the uptake was pronounced for the 50 nm particles, whereas the 200 nm NP were found to be accumulated on top of the cell layers to a higher extent. In contrast, neglectable particle internalization and translocation of 50 nm MSNs on Caco-2 mono-cultures were reported by Ye et al*.*^77^, where the majority of particles adhered on top of the cell layer. In the present study, no internalization into Caco-2 mono- or co-cultures was observed, whereas accumulation of MSN-PEG in organs of DSS-treated mice was found, thus pointing out the need for more advanced in vitro models to simulate intestinal resorption especially in cases of epithelial erosion and inflammation. Therefore, multi-layer co-cultures including macrophages and dendritic cells to study inflammatory responses and the role of macrophage infiltration on particle uptake and therapeutic effects^78,79^ or in vitro models with realistic 3D environments, for example organoids or tissue engineered architectures^80–83^, have been proposed in order to minimize in vivo studies. However, these models have drawbacks on their own, e.g. work-intensiveness and time-consumption, omitting high-throughput screening of components and parameters, and may be reserved for most promising candidates. Nevertheless, we demonstrated here the suitability of such epithelial models to simulate accumulation depending on the presence of mucus from which predictions regarding intestinal retention could eventually be made.

## Conclusions

In the present study, rod-shaped mesoporous silica nanoparticles were synthesized and further functionalized with PEG or HA. Upon oral administration of the particles to healthy mice and animals exhibiting DSS-induced colitis, the biodistribution in the GIT and organs over time was investigated via in vivo fluorescence imaging. In all cases, MSNs were found to be localized in all compartments of the GIT already 6 h after administration and to be still present after 14 h. While MSN-PEG remained abundant in the lower GIT of healthy animals over time, MSN-HA experienced continuous clearance. This was shown to be caused by differences in adherence of the polymer-functionalized particles to cell-based epithelial models in vitro. In the presence of a mucus layer, which acted as a diffusion barrier for MSN-HA, diminishing particle accumulation on the cell layers was observed. However, in mice with DSS-colitis, the MSN-HA particles were shown to accumulate in the lower compartments of the GIT, *i.e.* in the cecum and in the colon. Moreover, the possibility of particles to be taken up into the body and their biodistribution in other organs was examined. Particle resorption was found to be strongly dependent on animal health status and particle functionalization type. No MSNs were observed in organs of healthy mice, but intestinal inflammation promoted resorption of the particles, which was especially the case for MSN-PEG. Here, particles were found in the kidneys, the spleen and the heart/lungs 6 h post-administration, and signs of beginning blood clearance were found. Systemic uptake of MSNs could not be predicted by in vitro experiments where no internalization or permeation was observed, demonstrating the need for more ingenious in vitro systems to model the complex environment present in the inflamed GIT.

Overall, this work provides valuable information on particle accumulation and uptake under different conditions in vitro and in vivo. Although only semi-quantitative statements about particle biodistribution can be drawn from these results, this study serves as basis for further research to elucidate the quantitative portions of these MSN types present in the GIT under healthy and inflammatory conditions. The pronounced differences in particle accumulation dependent on the functionalization with PEG or HA are evident, underlining the high potential of MSNs as long-lasting delivery platform for oral drug delivery applications.

### Supplementary Information

Supplementary Figures.

## Data Availability

The datasets generated during and/or analyzed during the current study are available from the corresponding author on reasonable request.
