# Supplementary material for: Surface functionalization affects the retention and bio-distribution of orally administered mesoporous silica nanoparticles in a colitis mouse model
Source: Sci Rep. 2023 Nov 17;13:20175. doi: 10.1038/s41598-023-47445-6 (PMC10656483; doi:10.1038/s41598-023-47445-6)
Supplement: Supplementary file 1 — Supplementary Figures. [file 41598_2023_47445_MOESM1_ESM.docx]

**Supporting Information**


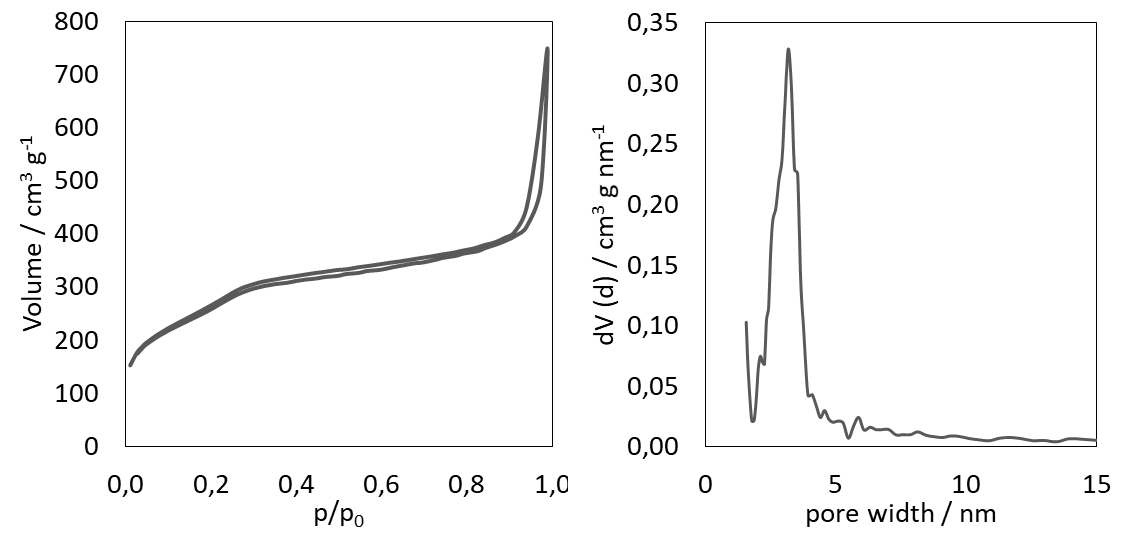


**Figure S1.** Nitrogen sorption isotherms of the calcined basic MSN-c (left) and corresponding pore size distributions calculated using the equilibrium NLDFT kernel developed for silica and a relative pressure range of p p_0_^-1^ = 0.9 (right).


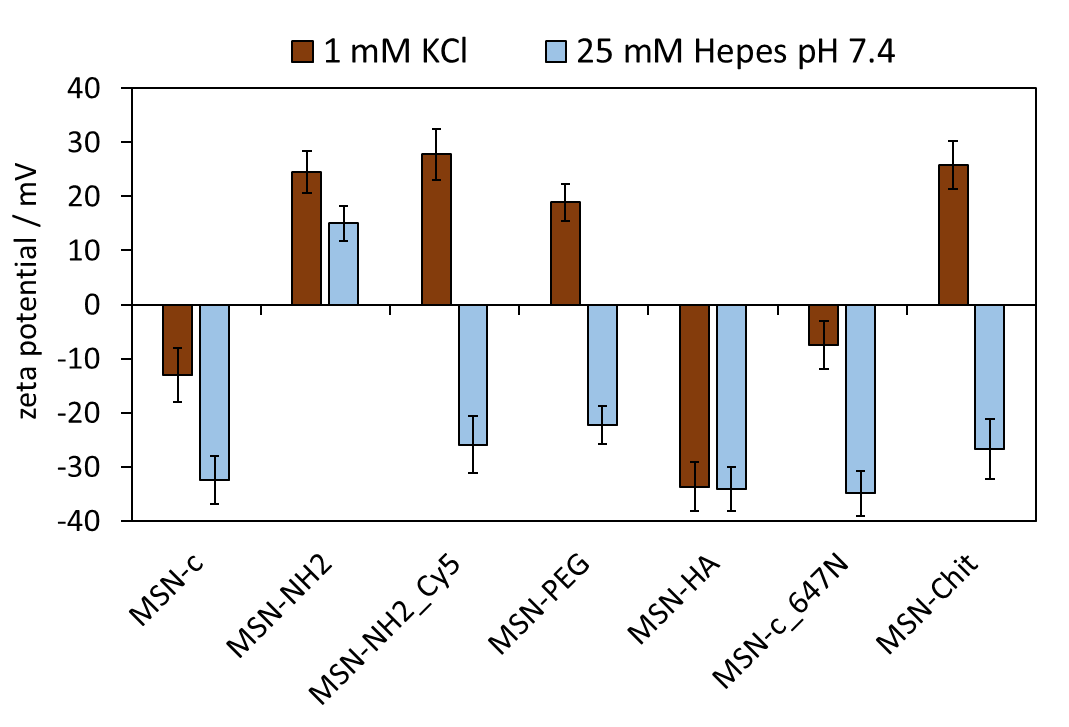


**Figure S2.** Zeta potential measurements of the synthesized particles at a concentration of 100 µg mL^-1^ in 25 mM HEPES buffer adjusted to pH 7.4 or non-buffered 1 mM KCl. The basic calcined MSN-c or amino-functionalized MSN-NH2 were labeled with the fluorescent dyes Cy5 or Atto647N, respectively, and further functionalized either with PEG and HA or chitosan. (mean ± SD, n = 3)


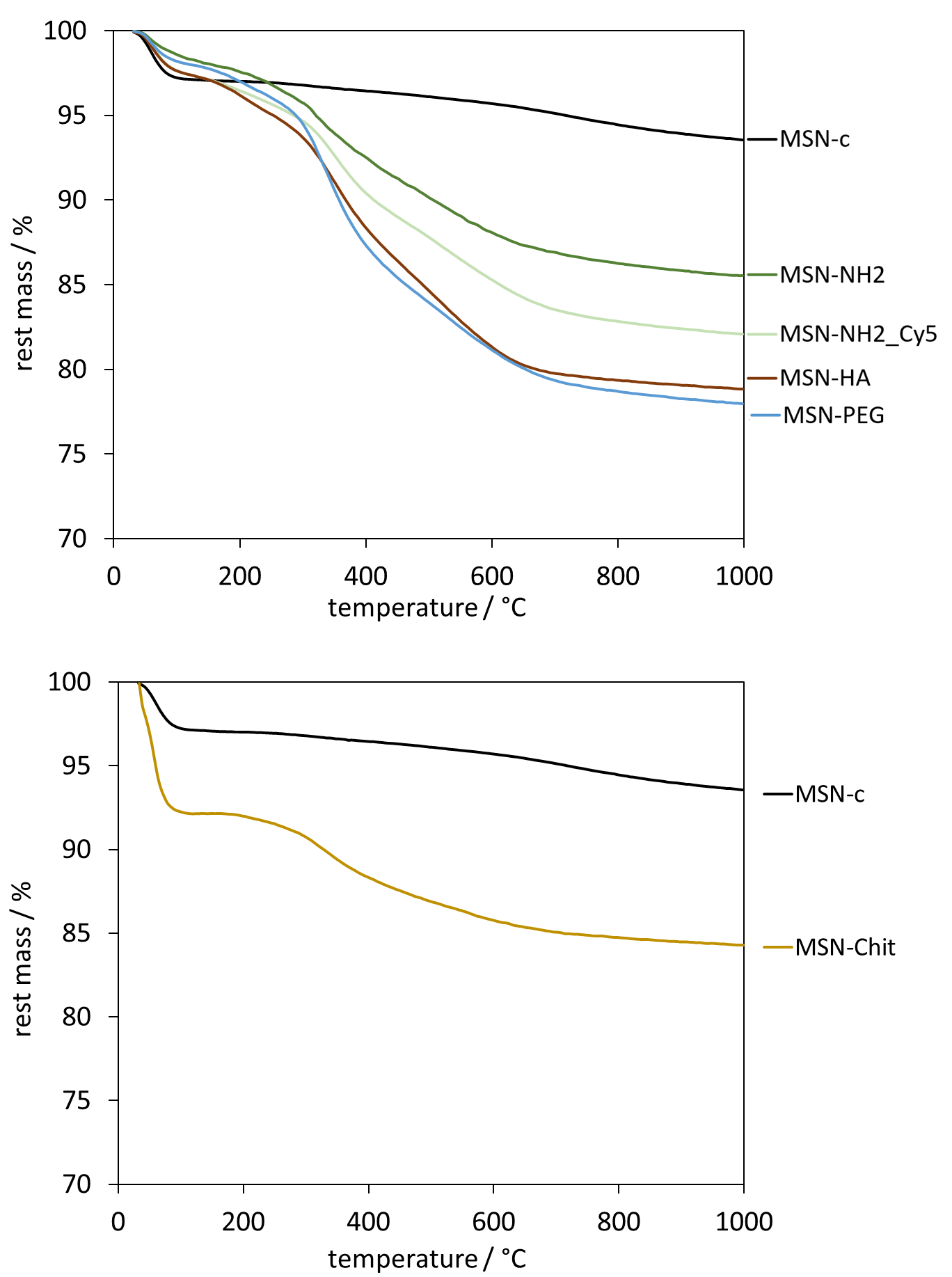


**Figure S3.** Thermogravimetric measurements of basic and functionalized MSNs.


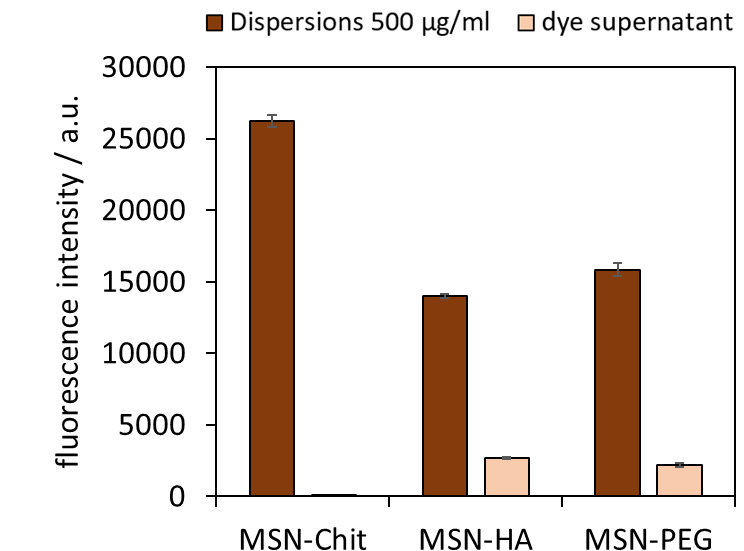


**Figure S4.** Fluorophore detachment from MSNs upon incubation under agitation in FaSSIF buffer for 14 h with a silica concentration of 500 µg mL^-1^ at 37°C is minor. Fluorescence intensities of supernatants were measured with an exitation and emission wavelength of 650 nm and 705 nm. Fluorescence intensities of the particle dispersions with a silica concentraton of 500 µg mL^-1^ prior incubation were measured as controls. (mean ± SD, n = 3)


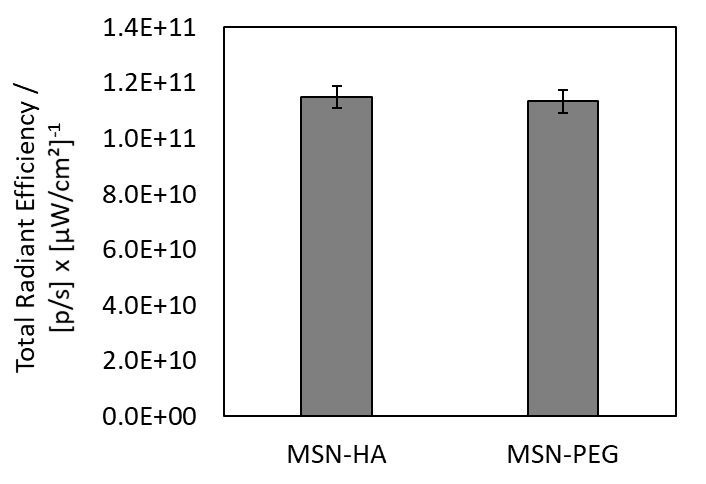


**Figure S5.** Total radiance efficiencies of particle dispersions at a concentration of 100 µg mL^-1^ measured via IVIS with an exitation and emission wavelength of 640 nm and 670 nm in a 96 well plates revealed no differences in fluorescence intensities. (mean ± SD, n = 3)


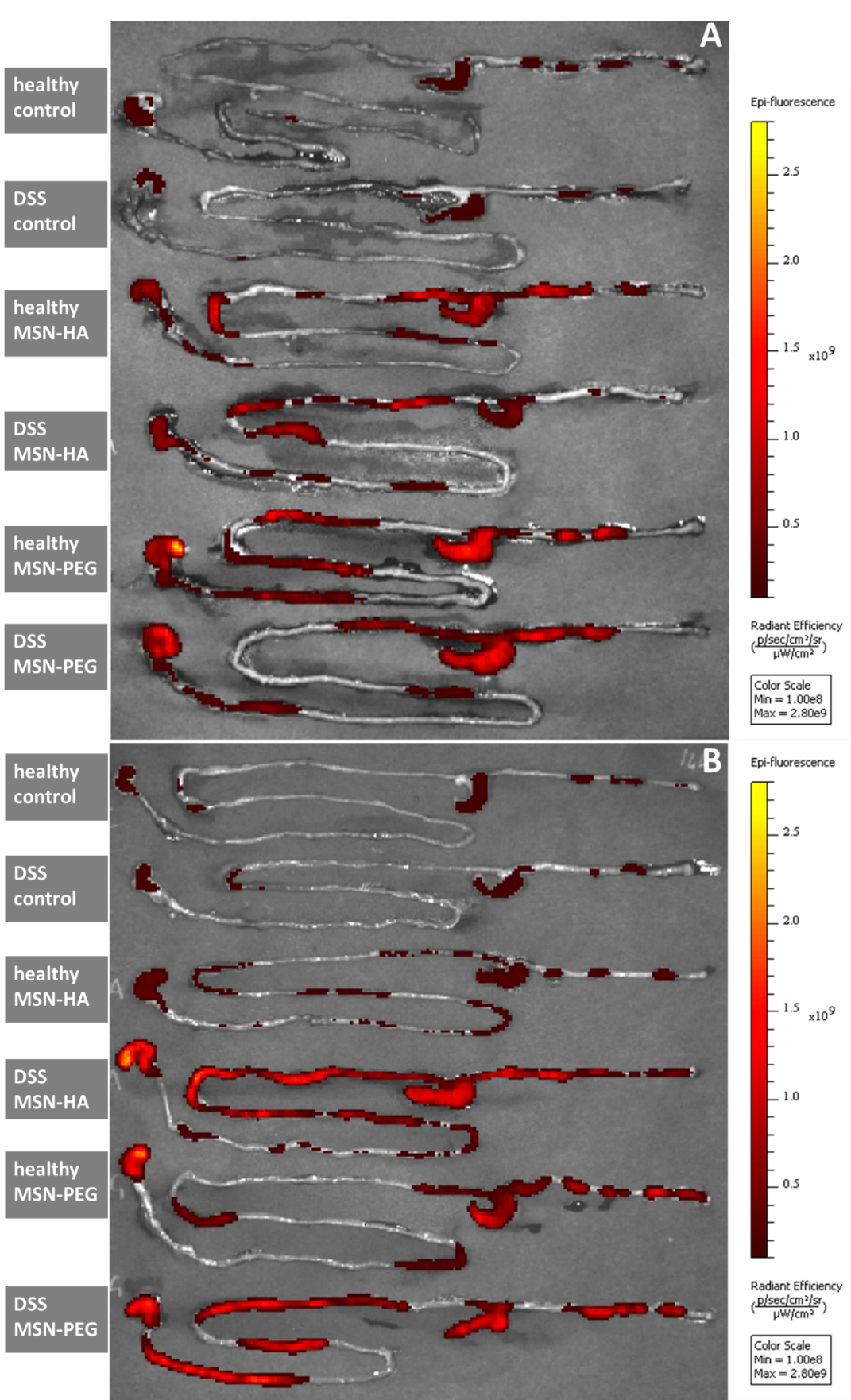


**Figure S6.** Exemplary fluorescence intensity measurements of excised GIT from mice measured via IVIS A) 6 h and B) 14 h after oral gavage of a single particle dose of 100 mg kg^-1^ (MSN-HA or MSN-PEG). Control animals received PBS only. Fluorescence intensities were measured with an excitation/emission wavelength of 640/670 nm. (n = 3)


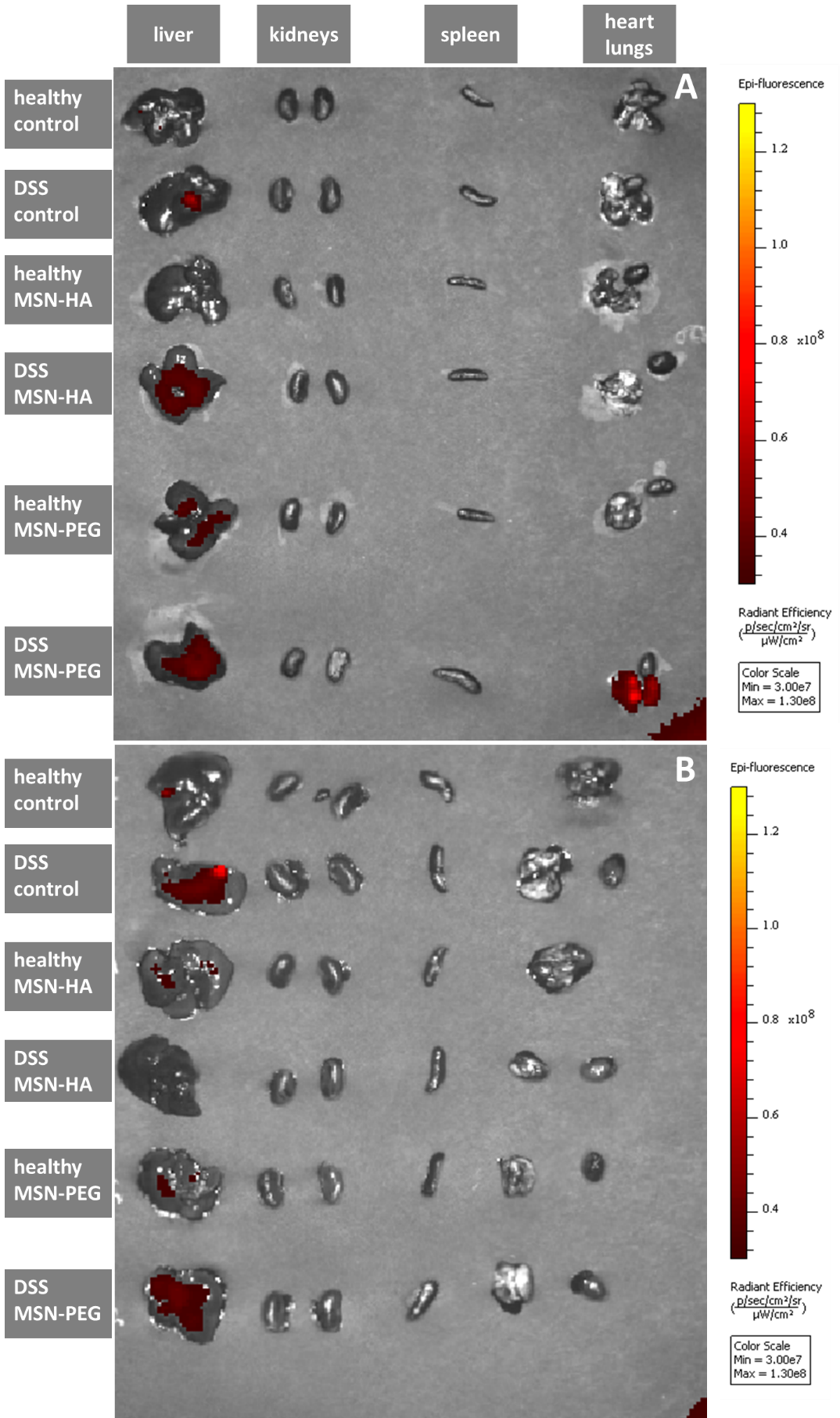


**Figure S7.** Exemplary fluorescence intensity measurements of excised organs from mice measured via IVIS A) 6 h and B) 14 h after oral gavage of a single particle dose of 100 mg kg^-1^ (MSN-HA or MSN-PEG). Control animals received PBS only. Fluorescence intensities were measured with an excitation/emission wavelength of 640/670 nm. (n = 3)


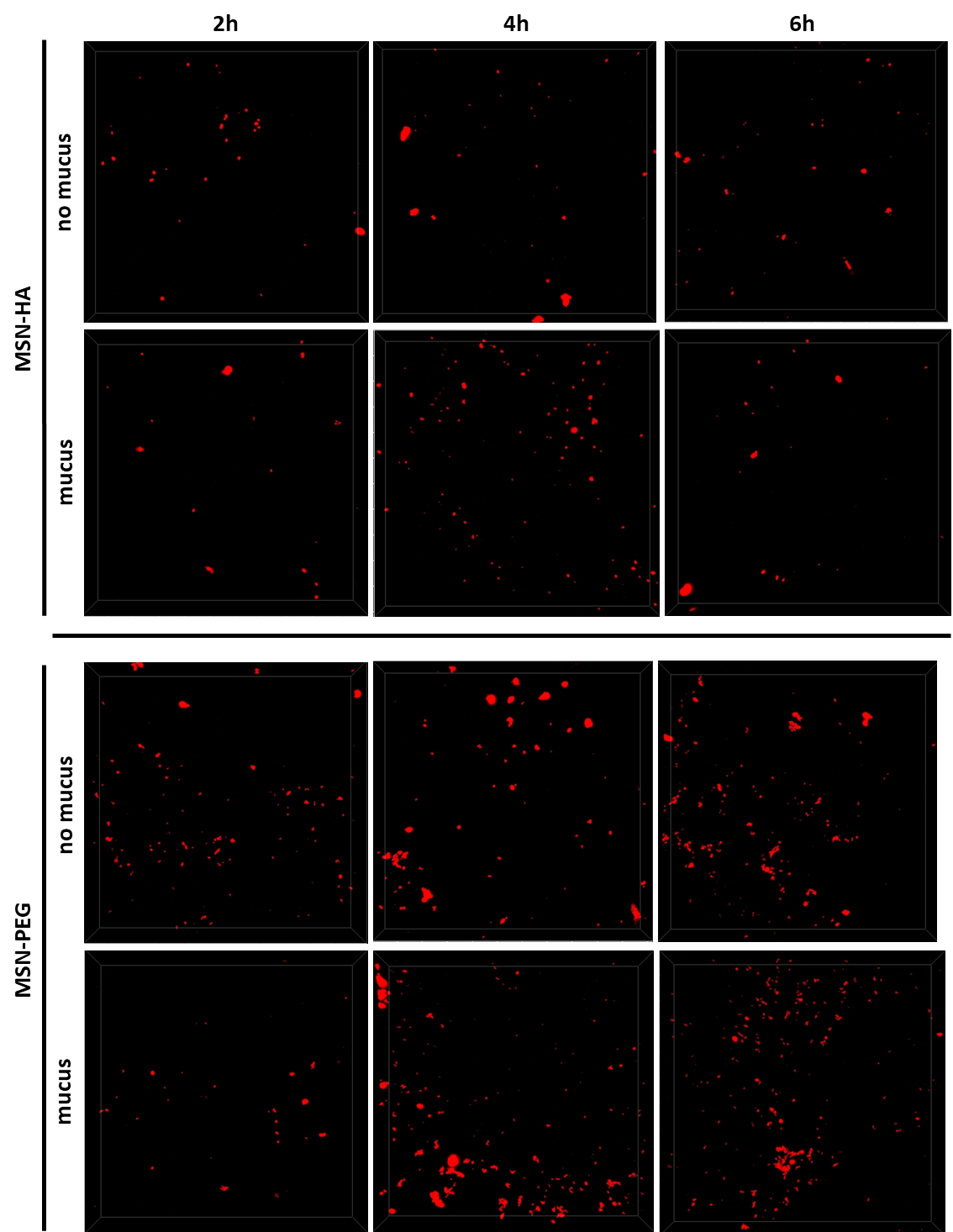


**Figure S8.** Representative confocal light scanning microscopy images of random areas of Caco-2/Raji (“no mucus”) or Caco-2/HT28/Raji (“mucus”) co-culture cell models after incubation with differently functionalized MSNs (red) at a concentration of 50 µg mL^-1^ for 2 h, 4 h and 6 h at 37°C (maximal intensity projections of on-top views of the epithelial cell model).


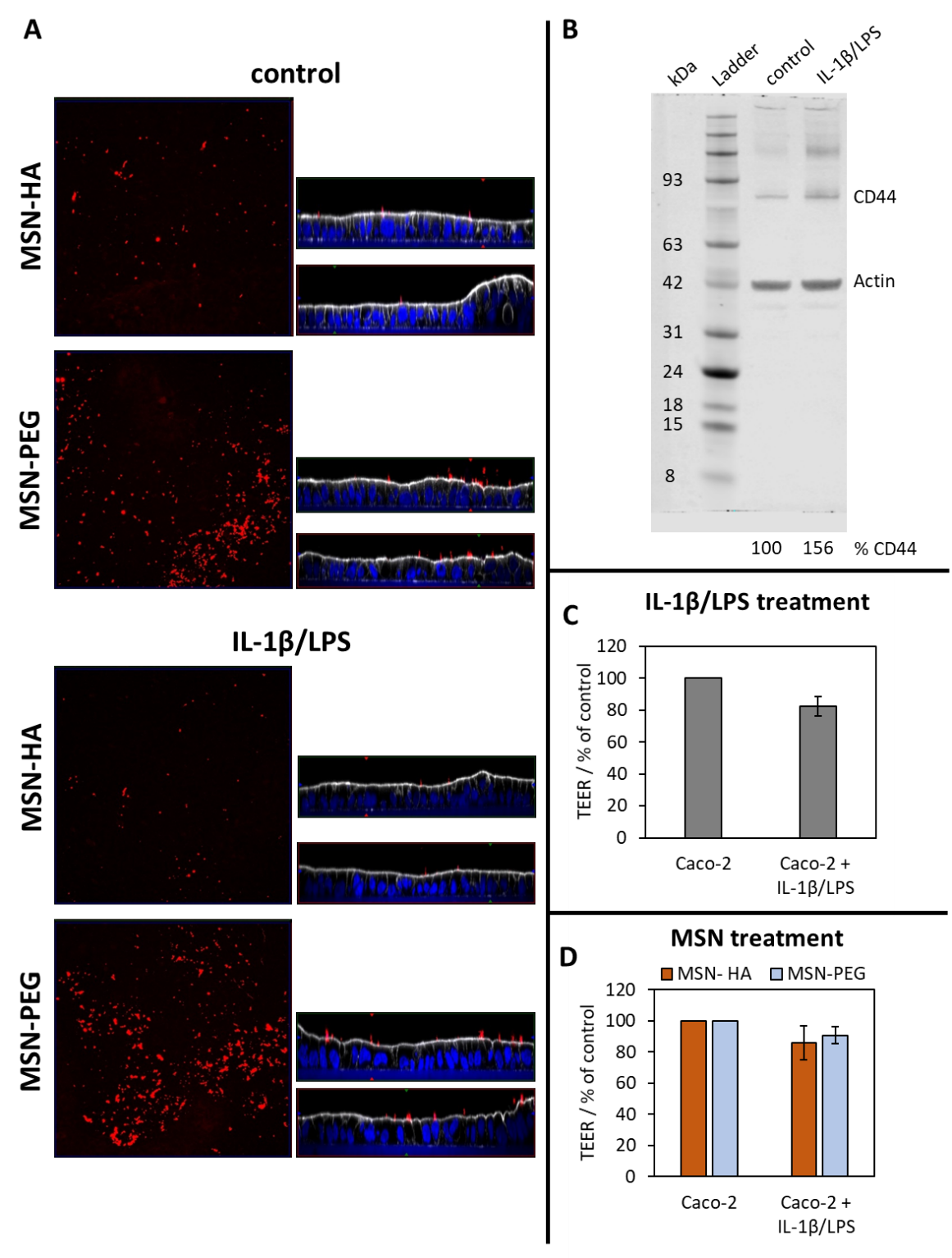


**Figure S9.** Caco-2/Raji co-cultures after treatment for 48 h at 37°C with ("IL-1β/LPS ") or without ("control") interleukin- 1β (IL-1β) and lipopolysaccharide (LPS) at a concentration of 50 ng mL^-1^ or 1000 ng mL^-1^, respectively. Subsequently, cell models were incubated with differently functionalized MSNs at a concentration of 50 µg mL^-1^ for 14 h at 37°C. A) Representative confocal light scanning microscopy images of random areas. Left column: maximal intensity projection of on-top views of the epithelial cell model. Right column: channel overlay of cross-sectional x/z and y/z side views. MSNs (red), cell membrane (white), nuclei (blue). B) Verification of CD44 overexpression after treatment with IL-1β and LPS via Western Blot analysis (full-length membrane). Treated cultures exhibited a CD44 expression 156 % higher than non-treated cultures (control). For a full, non-cropped image of the plot, the reader is referred to Figure S13. C) TEER measurements of model epitheliums after treatment with IL-1β and LPS. D) TEER measurements of epithelial cell models after incubation with MSN-HA and MSN-PEG. (mean ± SD, n = 3)


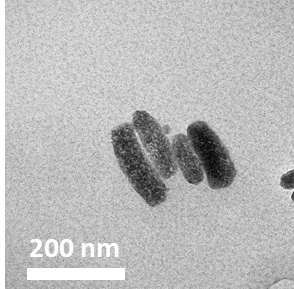


**Figure S10.** Transmission electron microscopy (200k magnification) of MSN-Chit after 14h incubation under agitation at 37 °C and a silica concentration of 500 µg mL^-1^ in FaSSIF buffer (pH 6.5).


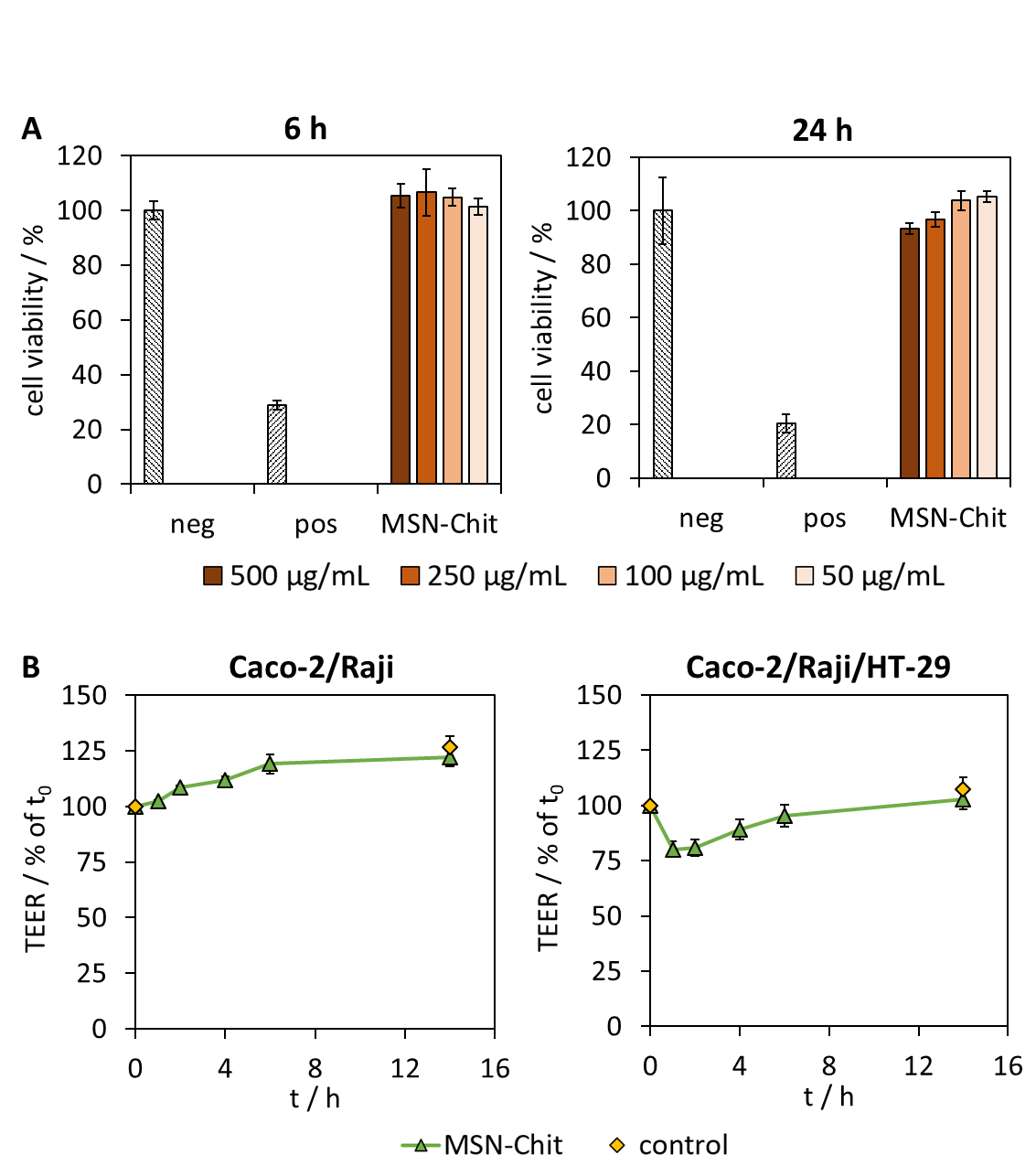


**Figure S11.** A) Cell viability of Caco-2 cells after incubation with MSN-Chit at varying concentrations for 6 h and 14 h as well as DMSO treated cells (pos) was measured with MTS-assay and was normalized on cells without treatment (neg). B) Measurement of the TEER of Caco-2 (left) and Caco-2/HT29 (right) epithelial cell models upon incubation with differently functionalized MSNs at a concentration of 50 µg mL^‑1^. TEER values were measured at distinct times and calculated as percentage of TEER before the addition of particles. (mean ± SD, n = 3)


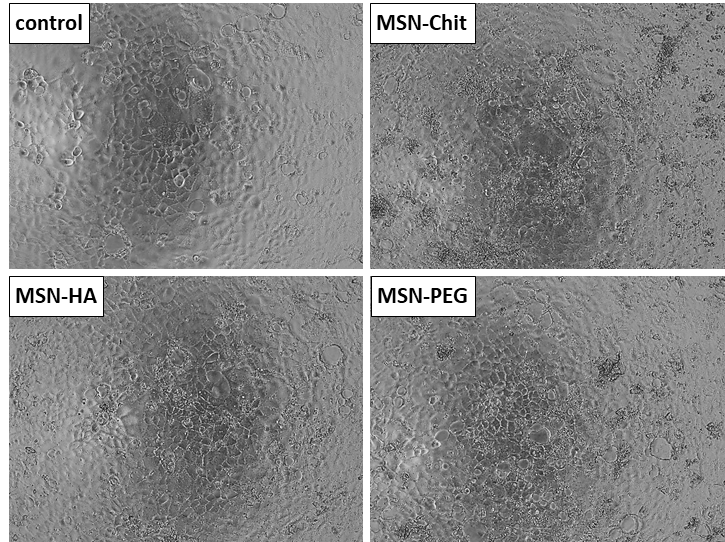


**Figure S12.** Representative light microscopy images of Caco-2 cells upon exposure of MSN-Chit, MSN-HA and MSN-PEG at a concentration of 500 µg mL^-1^ at 37°C for 24 h.


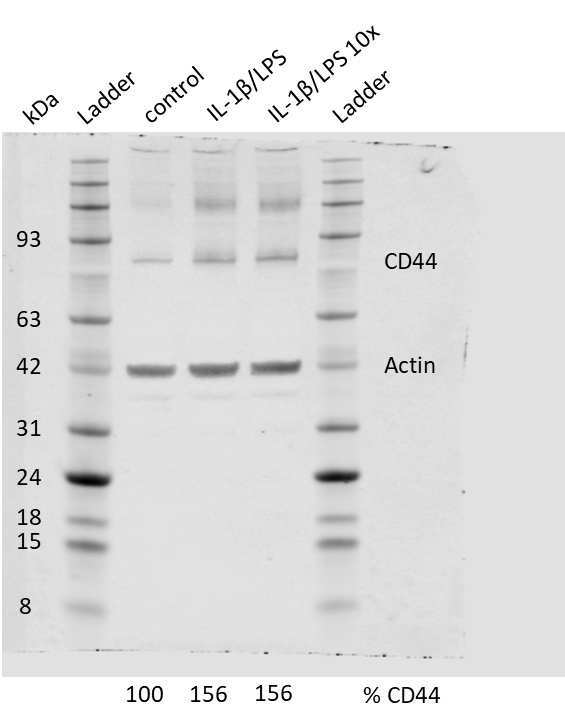


**Figure S13.** Full, non-cropped image of the Western Blot analysis from Figure S9. CD44 overexpression on Caco-2/Raji co-cultures was induced by treatment with ("IL-1β/LPS") or without ("control") interleukin-1β (IL-1β) and lipopolysaccharide (LPS) for 48 h at 37°C. IL-1β and LPS were applied at a concentration of 50 ng x ml^-1^ and 1000 ng x ml^-^1, respectively, and at a concentration increased by a factor of 10, i.e. 500 ng x ml^-1^ for IL-1β and 10000 ng x ml^-1^ for LPS ("IL-1β/LPS 10x"). However, no increase in CD44 expression was observed for the increased concentrations, which is why these cell cultures were omitted in the following experiments.
